# Smart Triboelectric Nanogenerators Based on Stimulus-Response Materials: From Intelligent Applications to Self-Powered Systems

**DOI:** 10.3390/nano13081316

**Published:** 2023-04-08

**Authors:** Xueqing Wang, Qinghao Qin, Yin Lu, Yajun Mi, Jiajing Meng, Zequan Zhao, Han Wu, Xia Cao, Ning Wang

**Affiliations:** 1 Center for Green Innovation, School of Mathematics and Physics, University of Science and Technology Beijing, Beijing 100083, China; 2 Beijing Institute of Nanoenergy and Nanosystems, Chinese Academy of Sciences, Beijing 100083, China; caoxia@binn.cas.cn

**Keywords:** smart materials, stimuli-responsive, triboelectric nanogenerator, energy conversion, self-powered system

## Abstract

Smart responsive materials can react to external stimuli via a reversible mechanism and can be directly combined with a triboelectric nanogenerator (TENG) to deliver various intelligent applications, such as sensors, actuators, robots, artificial muscles, and controlled drug delivery. Not only that, mechanical energy in the reversible response of innovative materials can be scavenged and transformed into decipherable electrical signals. Because of the high dependence of amplitude and frequency on environmental stimuli, self-powered intelligent systems may be thus built and present an immediate response to stress, electrical current, temperature, magnetic field, or even chemical compounds. This review summarizes the recent research progress of smart TENGs based on stimulus-response materials. After briefly introducing the working principle of TENG, we discuss the implementation of smart materials in TENGs with a classification of several sub-groups: shape-memory alloy, piezoelectric materials, magneto-rheological, and electro-rheological materials. While we focus on their design strategy and function collaboration, applications in robots, clinical treatment, and sensors are described in detail to show the versatility and promising future of smart TNEGs. In the end, challenges and outlooks in this field are highlighted, with an aim to promote the integration of varied advanced intelligent technologies into compact, diverse functional packages in a self-powered mode.

## 1. Introduction

Currently, the ever-growing need for versatile and efficient intelligent devices stimulates people’s intense research interest in integrating smart response materials with traditional electronics [1,2,3]. The original concept of smart materials stems from natural biological systems that follow a perception-reaction-learning mechanism. Presently, they are defined as materials that can respond to a variety of external stimuli or environmental changes. This is because smart materials can adjust their function and respond accordingly by rearranging their structure at the molecular level. They represent a family of innovative materials for understanding experiences with self-awareness and purposeful responses [4,5,6,7,8].

Historically, applications of functional smart materials with controllable shape or volume changes in response to external stimuli have been investigated in several frontier fields, such as sensors, actuators, optoelectronic devices, information storage, and biomedicine [9,10,11,12,13]. These external stimuli include chemical incentives (e.g., changes in concentration, humidity, pH) [14,15,16], mechanical stimuli (e.g., pressure, strain) [17,18], physical stimuli (e.g., light, sound, temperature, color) [19,20,21,22,23], and electromagnetic stimuli (e.g., electric, magnetic, charge injection) [24,25,26,27]. The unique and excellent responsive characteristics of smart materials enable them to be used specifically and accurately in certain applications, allowing them to perceive changes in the environment and adapt future smart polymers to similar situations and specific behaviors in specific applications [28,29,30].

A triboelectric nanogenerator (TENG) is a newly developed energy harvesting technology based on the coupling effect of contact charge and electrostatics [31,32]. It can convert almost any type of mechanical energy, such as vibration [33], water waves [34], wind [35], raindrops [36], and human motion [37], into electrical energy, and power various sensors. In principle, TENGs have a wide selection of materials, such as organic materials [27,38], polymers [39], hydrogels [40], and elastic rubbers [41,42], which can be directly used to manufacture TENG or serve as active component of TENG systems. Thus, smart materials can also be promising candidates combined with TENG devices and generate many multifunctional applications. The introduction of smart materials not only improves the performance of TENG in terms of material optimization, but also provides various applications, such as sensors [43,44], actuators [45], robots [46], artificial muscles [47], and controlled drug delivery [48]. Moreover, the mechanical energy in the reversible response of smart materials can be cleared and converted into electrical energy, which is highly dependent on the amplitude and frequency of environmental stimuli and can be digitized for the latter. Therefore, a self-powered smart system can be constructed, with broad applications in catalysis [49], sensors [50], drug/gene delivery [51,52], and self-assembly [53,54].

**Figure 1:**
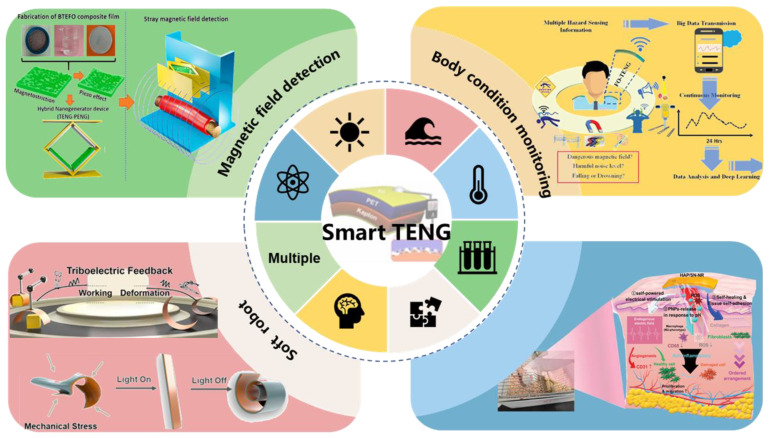
Diverse applications of TENGs that are based on multiple stimuli-responsive materials.

This review discusses recent advances in smart TENGs that are based on different stimulus-response materials (Figure 1). In the first part of the review, we give an in-depth introduction to the TENG, including stimuli based on electromagnetic drives and light responses. In subsequent sections, we highlight the application of smart response TENG in different research areas and future development prospects. Finally, the smart answer TENG discussion is summarized, and current research gaps and future research directions are suggested from a broader perspective.

## 2. Basic Principle and Working Modes of TENGs

**Figure 2:**
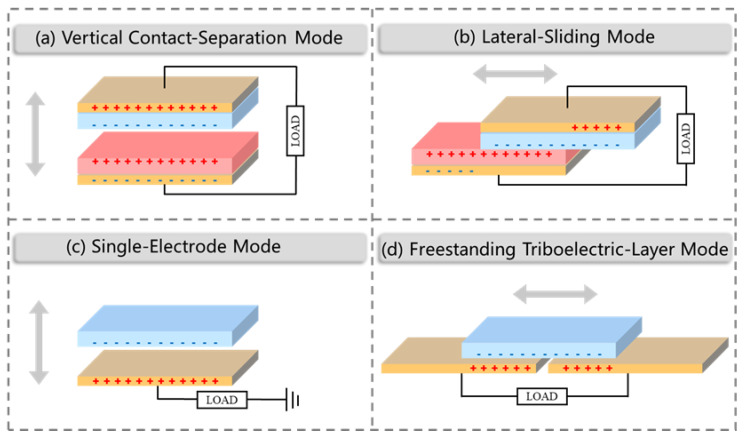
The basic principle of TENG.

TENG is capable of transforming mechanical energy from the surrounding environment into electrical power by utilizing a combination of triboelectrification and electrostatic induction effects [55,56]. Typically, this involves contacting two materials with differing charge affinities, causing electrostatic charges to build up on their surfaces until reaching saturation with repeated contact. These charges generate an electric field that drives the flow of electrons through an external load over time, ultimately converting mechanical energy into electrical energy [57,58]. TENGs can be categorized into four working modes(Figure 2): the vertical contact separation mode, which uses vertical polarization; the lateral sliding mode, utilizing lateral polarization from the relative movement between two media [59]; the single-electrode mode, which can harvest energy from a freely moving object without attaching conductive wires [60]; the independent triboelectric layer mode, which uses electrostatic induction between a pair of electrodes to generate electrical energy [61].

Vertical contact separation mode is the basic model of TENG, which involves the creation of opposite charges on the surfaces of two dielectric films with distinct electron affinity upon physical contact. When there is an appropriate gap between the two films, an electric potential difference occurs between the surfaces of the two dielectric films. If the films were connected through electrodes and external circuits, the free electrons in one electrode will flow to the other to balance the electrostatic field. Once the gap is closed, the potential difference caused by the friction charge disappears, and the induced electrons flow back. Regular contact and separation between the two materials drive rated electrons to flow back and forth between the two electrodes, resulting in an AC output in the external circuit. It is the basic model of TENG.

Lateral sliding mode involves relative movement in the direction parallel to the interface between two materials with opposite triboelectric polarities. When the materials are brought into contact, surface charge transfer occurs due to the triboelectrification effect. When the materials are separated by a distance, a potential difference is generated across two electrodes due to the effective dipole polarization created in parallel to the direction of the displacement.

Single electrode mode converts energy by combining dielectric materials with metal electrodes. When the charged dielectric approaches the grounded metal electrode, an induction charge is created in the metal plate to balance the electrostatic field. When the dielectric moves away from the metal plate, the current flows back to the ground. SE is the most convenient and simplest mode, which harvests the energy of moving objects through only a metal plate without the need for other dielectrics.

Independent triboelectric layer mode has high potential in collecting natural energy. The mode needs to place a dielectric layer on top of a pair of symmetrical electrodes that are approximately the same size as the moving object and maintain a small gap between the object and electrodes, an asymmetric charge distribution is generated via induction in the surrounding medium when the object approaches or departs from the electrodes. This occurs only if the object has been previously charged by a triboelectric process. The electrons flow between the two electrodes to balance the local potential distribution. As the object moves back and forth, the electrons oscillate between the paired electrodes and generate an AC output. This mode offers the advantage of harvesting energy from a moving object without requiring grounding, making the system more flexible.

The TENG’s open circuit voltage (*V_OC_*) can easily reach several thousand volts, while the short circuit current (*I_SC_*) is as low as a few microamperes. The output voltage of a TENG decreases as the load resistance decreases. Therefore, intelligent responsive materials can be used as the friction layer or electrode layer material of TENG to improve its output characteristics. Moreover, TENG can also be used to drive some intelligent and responsive materials and devices.

## 3. Classifications and Underlying Mechanisms of TENGs That Are Based on Smart Materials

The self-adaptability and self-actuation of smart materials are achieved through the autonomous movement of particles at the nanoscale. When stimulated by external factors, molecules and atoms will aggregate, align, and orient themselves to provide the optimal response to the external stimuli [62,63]. If combined with TENG, the system may deliver various intelligent applications, such as sensors [64], actuators [65], robots [66], artificial muscles [67], and controlled drug delivery [68] with immediate response to stress, electrical current, temperature, magnetic field, or even chemical compounds. In this chapter, we introduce the combination of different types of smart materials with TENG based on their principles and mechanisms. In Table 1, we list the synthesis methods, properties, and application comparison of TENGs combined with different types of smart materials. Specifically, we present the working mechanism of the smart material in shape-memory, self-healing, and other response polymers, discuss the basic principle of the stimulus-response characteristics, and illustrate how the smart fabric can be combined with TENG to obtain better output performance and diverse application scenarios.

### 3.1. Magnetic Responsive Materials

In an external magnetic field, flexible materials embedded with magnetic particles can produce a series of deformations, such as stretching, deformation, and expansion. A controllable magnetic field can control these deformations of soft magnetic materials because the interaction between electrons creates a solid molecular lot in ferromagnetic materials. Ferromagnetic materials, in the process of spontaneous magnetization, will produce a small magnetization in different directions to reduce the magnetostatic energy, resulting in saturation magnetization. This is called the magnetic domain [87,88].

**Figure 3:**
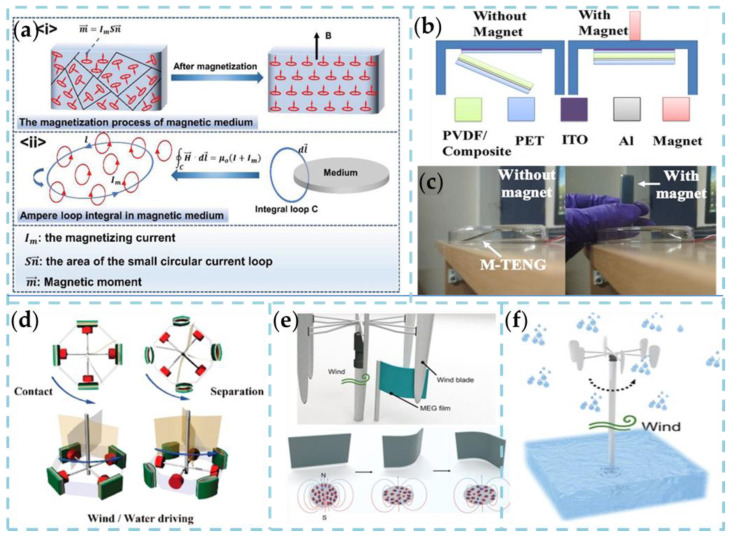
(**a**) The proposed enhancement mechanism of ferromagnetic-based S-TENG []. Copyright 2021, Wiley-VCH. (**b**,**c**) Schematic of M-TENG structure, with and without magnet and response of magnetic nanocomposite layer with and without magnet [69]. Copyright 2019, AMER CHEMICAL SOC. (**d**) Demonstration and schematic illustration of magnetic-assisted noncontact TENG for harvesting energy from wind and water flow (four devices arranged with perpendicular angles [70]. Copyright 2016, ELSEVIER. (**e**) A further detailed schematic of the zoomed-in wind blade and the correct corresponding wind rotation. The MEG can be seen on the right in its compressed state. (**f**) Schematic demonstration of the wind initiating the process of the generator [71]. Copyright 2022, WILEY-VCH VERLAG GMBH.

Thus, when a soft magnetic material is affected by the strength of an external magnetic field, the functional magnetic domains will align in the direction of the area. Magnetic soft materials are considered ideal alternative materials for specific space components due to their responsiveness, ease of handling, and self-assembly. At present, the research field and application range of intelligent magnetic response devices is less diverse than other types of response materials. In most cases, they are combined with flexible polymer materials and magnetically driven, employing unidirectional magnetization. Further investigation shows that the magnetic directions of the domains are randomly distributed due to the irregularity of the thermal motion, which causes the magnetic moments between the disciplines to cancel each other due to the opposite directions. Thus, in an external magnetic field with a strength of 0, the entire ferromagnet does not show magnetization. Once the external magnetic field drives the magnetic moment, it tends to coincide with the direction of the external magnetic field. In this dynamic process, an induced magnetic field is formed, and a magnetized current is generated (Figure 3a). Li et al. applied magnetized wind generated by a magnetic field to ferromagnets, which improved the electrical output performance and energy conversion efficiency of TENG and opened up a breakthrough direction for studying the output characteristics of TENG [89].

The magnetic nanocomposites used to create the Magnetic Response Triboelectric Nanogenerator (TENG) offer a unique benefit beyond the additional driving force provided by the magnetizing current. Specifically, the solid magnetic response of the material enables the external magnetic field to facilitate the contact and separation of the TENG’s layers, without requiring any additional mechanical force. Therefore, it can work in non-contact mode to prevent the device from coming into direct contact with external mechanical shocks that could damage the electrodes and triboelectric layer. Bushara et al. synthesized γ-Fe_2_O_3_/PVDF transparent nanocomposites as friction layer materials for TENG by spinning coating method, thus producing TENG devices with flexibility and stretchability advantages and stability [69]. In the preparation process, PET material was used as the positive triboelectric layer material, and on one of the substrates, the spun γ-Fe_2_O_3_/PVDF was used as the negative triboelectric material. The two are connected face to face to a curved mylar film, and the spacing between them can be periodically changed by externally applied forces and create a potential difference. As shown in Figure 3b,c, The TENG moves vertically in response to the attraction of the magnet. When the magnet is near the device, the magnetic nanocomposite is near the PET’s upper surface. When the magnet is removed from above the device, the PET layer and the nanocomposite layer separate due to gravity. The electrical output performance of the magnetic drive TENG in non-contact mode is 0.8 μA and 90 V, respectively. In addition, the output characteristics can be further optimized by changing the nanocomposite’s magnetized flux density or the NP embedment’s mass fraction.

In the past few years, TENG has been studied to capture wind and blue energy. Environmental factors for the outdoor energy harvesting process of the TENG in an open environment, such as gas composition, especially humidity, have a significant impact on the performance of the TENG. To adapt to wind energy and blue energy harvesting in high-modesty environments, Huang et al. proposed a magnetic-assisted non-contact TENG in which the device consists of two parts: a magnetic response layer (PDMS/FeCoNi composite/PET/Al/PDMS) and a non-magnetic response layer (Al/Ni fabric/PET) [70]. As shown in Figure 3d, wind or water flow can drive the rotation of the entire device, directly leading to contact separation of the magnetically assisted non-contact TENG. As the magnet rotates toward the device, the magnetic response layer is absorbed, causing the PDMS film to come into contact with the Al/Ni fabric. In contrast, the PDMS film and Al/Ni fabric separate as the magnet rotates away from the device. The blue energy can be harvested and converted into electrical energy by a contact separation between the magnetic and non-magnetic responsive layers. Obviously, the higher wind speed makes the panel spin faster, resulting in a higher frequency of contact separation between the magnetic and non-magnetic auxiliary layers.

Recently, Zhao et al. developed a magnetic elastomer generator that can change the magnetic field in a soft system through wind-induced mechanical deformation. Additionally, it can convert wind energy into electrical energy through electromagnetic induction in any direction (Figure 3f) [71]. As shown in Figure 3e, the magnetic-elastic generator is composed of magnetic particles and a polymer matrix with Young’s modulus of 980.9 kPa, which has suitable mechanical tensile properties. The results indicate that a high current density of 1.17 mA cm^−2^ and an extremely low internal impedance of 68 Ω are obtained at 266 r/min and driven by ambient rain-blown wind. The system can decompose water to produce hydrogen at a rate of 7.5 × 10^2^ mL h^−1^ at a wind speed of 20 m s^−1^. In other words, this work brings a whole new platform technology to the wind harvesting community.

### 3.2. Optical Responsive Materials

Light stimulation is a fundamental and straightforward approach for investigating single stimulus responses, offering fast response times, high driving performance change rates, and excellent stability [90,91,92]. In particular, photochromic molecules are crucial components of light-responsive materials, capable of capturing optical signals and converting them into valuable properties that can drive changes in shape and structure, as well as exhibit macroscopic characteristics [93]. Integrating photochromic materials into TENG systems enables the expansion of self-powered sensing applications and propels the development of TENG towards higher precision [94].

**Figure 4:**
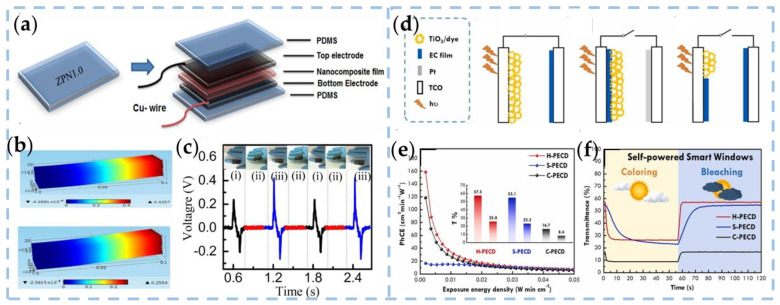
(**a**) Schematic diagram of the PCNG layer by layer. (**b**) Simulation of piezo potential distribution within PCNG during upward and downward bent states. (**c**) The embedded PCNG response synchronized with a human finger gesture, for example, (i) upward, (ii) relaxed state, and (iii) downward movements. [72]. Copyright 2016, IOP Publishing Ltd. (**d**) Classification of PECDs: (i) Separated type PECD (S-PECD); (ii) Combined type PECD(C-PECD); (iii) Hybrid type PECD (H-PECD) (**e**) Plot of PhCE versus exposure energy density of different types of PECDs. Inset: Transmittance for the bleached and colored states of corresponding PECDs; (**f**) Dynamic transmittance responses of different types of PECDs under illumination and dark for simulating the functional condition of corresponding self-powered smart windows under sunny and cloudy conditions [73]. Copyright 2021, ELSEVIER.

Santanu Jana et al. have produced a wearable polymer composite NG (PCNG), which facilitates the formation of γ crystalline phases by adding ZNO-NPs to PVDF films [72] (Figure 4a). PCNG delivers an open-circuit voltage of up to 28 V and a short-circuit current of 450 nA by simple repetitive human finger application (at a pressure amplitude of 8.43 kPa) (Figure 4c). Figure 4b shows experimental observations from the COMSOL multi-physical field software that validate the simulated potentials of PCNG attached to fingers in the upward- and downward-bending states. More importantly, when a perceptive response is generated by PCNG, where ZNO-NPs can be used as an optical probe, near-blue emission is observed from the photoluminescence spectrum.

The original model of solar-driven photo electrochromic devices (PECDs) is a hybrid system of dye-sensitized solar cells (DSSCs) and electrochromic devices (ECDs), which is a potential application for convenient self-powered applications. According to the structure design, PECDs can be divided into two classical types: separated photochromic devices (S-PECD) and combined photochromic devices (C-PECD), as shown in Figure 4d(i),(ii). In order to further improve the optical contrast and reduce the response time, a novel hybrid photochromic device (H-PECD) was proposed by Cheng et al. [73]. The novel H-PECD structure comprises a photoelectrode coated with an electro-chromic conducting polymer layer, which operates independently and exhibits exceptional speed and optical variation during coloring and bleaching under diverse weather conditions. This performance is comparable to conventional ECDs while circumventing the necessity for an additional power supply or intricate wiring.

### 3.3. Hygro-Responsive Materials

When the external environment’s relative humidity (RH) changes, the humidity-sensitive material can produce reversible expansion and shrinkage phenomena. Meanwhile, it can convert chemical potential energy in water into mechanical energy. Therefore, changing humidity conditions can provide the driving force for the TENG to switch to the extreme environment. The moisture-sensitive TENG can effectively capture energy from wind and raindrops. On the one hand, the device can optimize the working mode and automatically switch the working structure without outside intervention, forming a wholly self-powered intelligent system. On the other hand, the particular advantages of TENGs, such as fast response speed and high output voltage, can be fully utilized. Therefore, introducing hygro-responsive materials into the TENG research route could generate some attractive diversified applications in personal health monitoring or meteorology.

**Figure 5:**
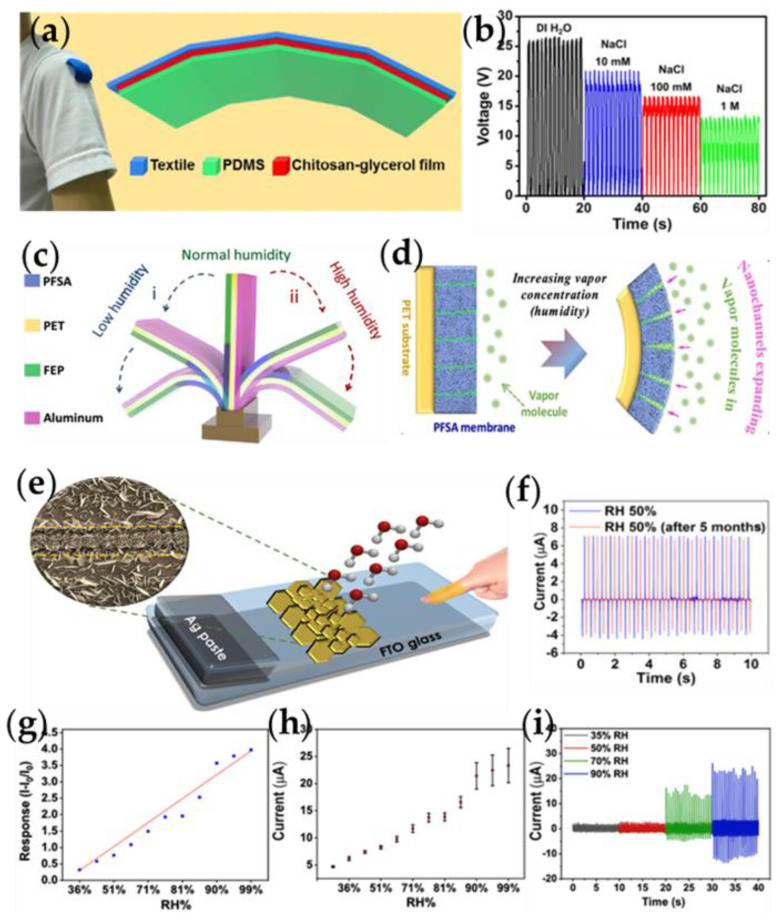
(**a**) Components of the self-powered sweat sensor. (**b**) The output voltage of the self-powered sweat sensor when detecting clothes worn with various concentrations of NaCl solution adsorbed [74]. Copyright 2018, ELSEVIER. (**c**) Schematic illustration of the humidity-responsive TENG based on the moisture/vapor-driven smart material (PFSA). (**d**) Deformation mechanism of the TENG blade in response to changed humidity [75]. Copyright 2019, AMER CHEMICAL SOC. (**e**) Schematic diagram of a self-powered dynamic response humidity sensor based on SnS_2_. (**f**) Current increases by varying the level of RH from 30 to 99% at room temperature, (**g**) sensor response by increasing RH at room temperature, (**h**) Dynamic responses of the self-powered SnS_2_-based humidity sensor by exposing to different RH based on TENG source power (**i**) Stability of sensor responses to 50% humidity over 5 months [76]. Copyright 2022, AMER CHEMICAL SOC.

Jao et al. have developed a triboelectric nanogenerator based on chitosan-glycerol thin films (C-TENG), which have resistance, environmental stability, and shape adaptability [74]. The output characteristics of the manufactured C-TENG are very stable under various humidity conditions. More importantly, the C-TENG can be combined with our functional textiles to enable a self-powered sensor (Figure 5a) to detect humidity, sweat, and gait phases. As shown in Figure 5b, with the increased NaCl concentration, the output voltage of the sweat sensor decreases from 26 V to 12.5 V. Because of this output tendency, NaCl sensors can be implemented. Since the concentration of NaCl is an essential indicator for people to monitor their physical condition during exercise, especially for high-intensity or long-term exercise, C-TENG can be used to detect the concentration in sweat.

Humidity sensing is a well-studied field of smart response. The vapor-absorbing materials used to make moisture-responsive actuators usually have porous or fibrous structures designed to allow better passage of water molecules, and the change in water molecule content will produce internal stress, thus causing spontaneous deformation. To achieve a self-actuated TENG, a steam-driven actuator is an ideal device for coupling with the TENG. Ren et al. demonstrated a wet-responsive TENG for vapor-driven materials based on perfluorinated sulfonic acid (PFSA) ionomers (Figure 5c) [75]. As shown in Figure 5d, at relatively high (or low) concentrations of PFSA membranes, water molecules can be absorbed (or desorbed) by these nanochannels, which gradually expand (or contract) to generate internal stresses and drive TENG leaf deformation. This self-powered sensor can be used to detect vapor concentration changes and monitor vapor leakage.

In addition, materials with two-dimensional (2D) structures have significant advantages in many areas of sensors. SnS_2_ has the most exciting design among two-dimensional (2D) transition metal disulfide (TMD) materials, consisting of a tightly packed sheet of tin atoms sandwiched between two sheets of sulfur atoms. It is worth noting that SnS_2_ material has high carrier mobility compared with other TMD material structures. The essential method to obtain SnS_2_ with different morphologies is chemical vapor deposition (CVD). Through this method, SnS_2_ can acquire a large number of available active sites, which is the critical parameter to realize gas or humidity sensing. Leyla et al. made a humidity sensor based on FTO/Kapton CS-TENG using SnS_2_ (Figure 5e) [76]. Figure 5g,h shows the current change and response of the SnS_2_-based humidity sensor at 30~90% RH. The SnS_2_-based self-powered humidity sensor has a downward trend in resistance when the device is exposed to the humidity range of 30–99% RH, which matches the active region of the FTO/Kapton TENG. Figure 5i shows the dynamic response of the self-powered TENG at room temperature at 30–70% RH. The output stability tested at 50%RH for more than five months is shown in Figure 5f. It can be seen that humidity sensing based on the TENG system can be realized in the fresh sensing layer. After 5 months, the SnS_2_-based self-powered sensor responds to humidity to the same degree, with a stable output current of about 7.6 μA, similar to the magnitude of the output current at 50% RH.

### 3.4. Temperature-Responsive Material

An important property of temperature-responsive materials is the low critical solution temperature (LCST). LCSTS are mainly found in polymer-based materials, and the polymer will dissolve into micelles in various proportions at temperatures below the LCST. In contrast, at temperatures above the LCTS, it will form separate phases [95,96]. Intelligent temperature response TENG is vital for energy harvesting and application expansion. The current working environment of electronic devices is mostly room temperature. In fact, temperature also has a non-negligible influence on the output performance of TENG, which limits their applicability significantly. Combining the temperature-sensitive material with the TENG can not only directly and accurately judge the real-time temperature through the output signal but also serve as a probe to reflect the ambient state at different temperatures.

**Figure 6:**
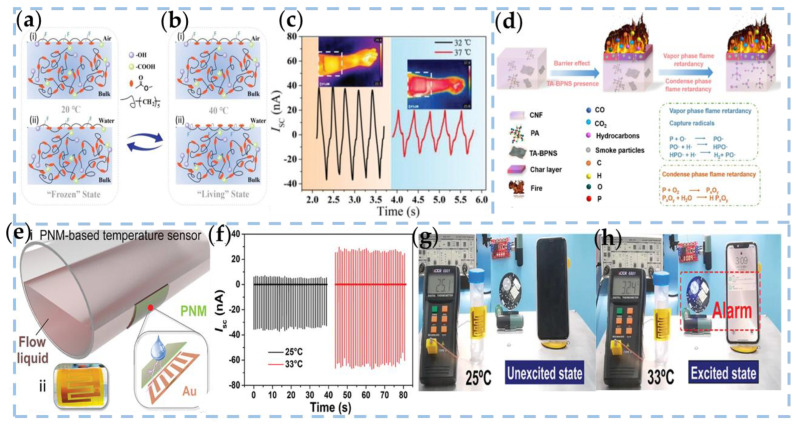
Conformation of PCL chains at (i) air–polymer interface and (ii) water–polymer interface at (**a**) 20 °C and (**b**) 40 °C. (**c**) Wearable liquid–solid TENG is used for detecting human body temperature [77]. Copyright 2021, WILEY-VCH. (**d**) Flame-retardant mechanism illustration of CNF-BP-PA film [78]. Copyright 2022, ELSEVIER SCIENCE SA. (**e**) Schematic diagram of the PNM-based temperature sensor with designed structure, (**f**) detected short-circuit current (*I_SC_*) when droplets impact onto the surface of PNM under 25 and 33 °C. (**g**,**h**) The photographs show the “unexcited” and “excited” state of the warning sensor under 25 and 33 °C, respectively [79]. Copyright 2022, WILEY-VCH VERLAG GMBH.

Li et al. utilized the intelligent temperature-regulating behavior of tribological properties to propose a poly(ε-caprolactone) (PCL)-based liquid–solid TENG. The PCL material comprises a hydrophobic backbone [-(CH2)5], hydrophilic end groups (-COOH, -OH), and amphiphilic ester groups, rendering it thermally responsive [77]. As a heterogeneous polymer, the surface of PCL will recombine when the temperature changes, even when the polymer is in a glassy state. Under relatively low temperatures (20 °C), the PCL chain migrates slowly, resembling the “frozen state” (Figure 6a), which is the thermodynamically unstable state. As the temperature increases (40 °C), the PCL chain transitions to an “active state”, and most of the hydrophobic groups at the interface are eventually replaced by hydrophilic groups (Figure 6b), reaching a state of thermodynamic equilibrium. As shown in Figure 6c, the wearable-device-based PCL liquid–solid TENG is designed to detect abnormal body temperature to monitor human health.

Recently, most TENGs based on biologically derived natural materials have been reported. Nanocellulose is a rich raw biomolecular material, which is renewable, bio-degradable, and highly biocompatible. Wang et al. developed a flexible and biodegradable flame retardant friction nanogenerator (FR-TENG) using cellulose nanofibrils (CNF) and phosphorous flame retardants for fire monitoring and warning purposes [78]. Tannic acid (TA) modified BPNS (TA-BPNs), and PA were added to CNF as non-toxic co-flame retardants as triboelectric layers (CNF-BP-PA films) and silver nanowires (AgNWs) as conductive layers. Compared to pure CNF films, CNF-BP-PA/AgNWs films exhibited excellent fire resistance and flame retardation, with a thermal index of 3779.16 K, indicating the film’s sensitivity to temperature. Figure 6d shows the combustion mechanism of FR-TENG based on CNF-BP-PA composite membrane. The BPNS of composite CNF-BP-PA films can be used as the flame-retardant mechanism of condensing and gas phases to achieve effective flame-retardant performance. At the initial stage of combustion, the layered structure of TA-BPNS can act as a physical barrier to prevent the transfer of pyrolytic products to the flame and simultaneously inhibit continuous combustion by generating heat. Subsequently, for the condensation phase flame retardant mechanism, BP will be oxidized to P_2_O_5_, promoting the dehydration and carbonization of the polymer, forming a protective carbon layer. In addition, P_2_O_5_ traps water molecules and includes a series of phosphoric acids that coated the surface of the material, blocking oxygen and heat from the outside world. The feasibility of FR-TENG in temperature sensing and its application in fire alarms are proven.

N-isopropyl acrylamide (NIPAM) has a thermal phase transition behavior and intelligent adjustable properties as a biologically compatible and temperature-sensitive material. Poly (n-isopropyl acrylamide) (PNIPAM) is water soluble at room temperature and has a low critical solution temperature (LCST) of 32 °C [97,98]. By changing the external temperature, it can undergo a phase transition from curl to the ball. Feng et al. prepared the temperature-sensitive NIPAM by free radical polymerization and spin coating and introduced it into L-S TENGs [79]. Due to its thermal properties, the main groups and surface wettability vary with temperature. On this basis, in order to demonstrate the practicality of the PNM-based L-S TENG, an alarm device was designed to monitor fluid pipe temperatures exceeding 30 °C. The assembly of the monitoring device is shown in Figure 6e. In the temperature sensor design, the tribological energy generated by water droplets flowing over the surface of PNM at different temperatures is the key to triggering the alarm. Figure 6f shows the *I_sc_* at different temperatures when the droplet impinges on the PNM surface, and the tilt angle is 40°. At 25 °C and 33 °C, a droplet sliding on the device can obtain an output of 35 and 64 nA, respectively. According to the output difference, the threshold *I_sc_* is set to trigger the wireless alarm sensor. As shown in Figure 6g,h, when the droplet touches the PNM surface at 33 °C, the transmitter sends out a wireless signal; at the same time, the wireless receiver receives the corresponding password and wakes up the alarm, and the mobile phone receives the alarm information in real-time. The size and frequency of the droplet can be obtained from the triboelectric output, and corresponding measures can be taken. Since the LCST of PNM is close to the physiological temperature of the human body, there will be more scenarios for applying PNM temperature sensing in the future.

### 3.5. pH-Responsive Materials

pH-responsive materials have attracted much attention from academia and researchers in the past few decades. pH-responsive polymers are materials that respond sensitively to the pH of solution through changes in structural properties such as solubility, surface activity, configuration, and chain structure formation [99,100]. Polymer-based materials were widely used as pH response materials during their development because of their structurally weak acid/primary groups. Significant changes in pH value can lead to swelling/deswelling of polymers, dissolution/precipitation of polymer chains, or the effect of hydrophobic/hydrophilic surface characteristics on changes in surface properties.

**Figure 7:**
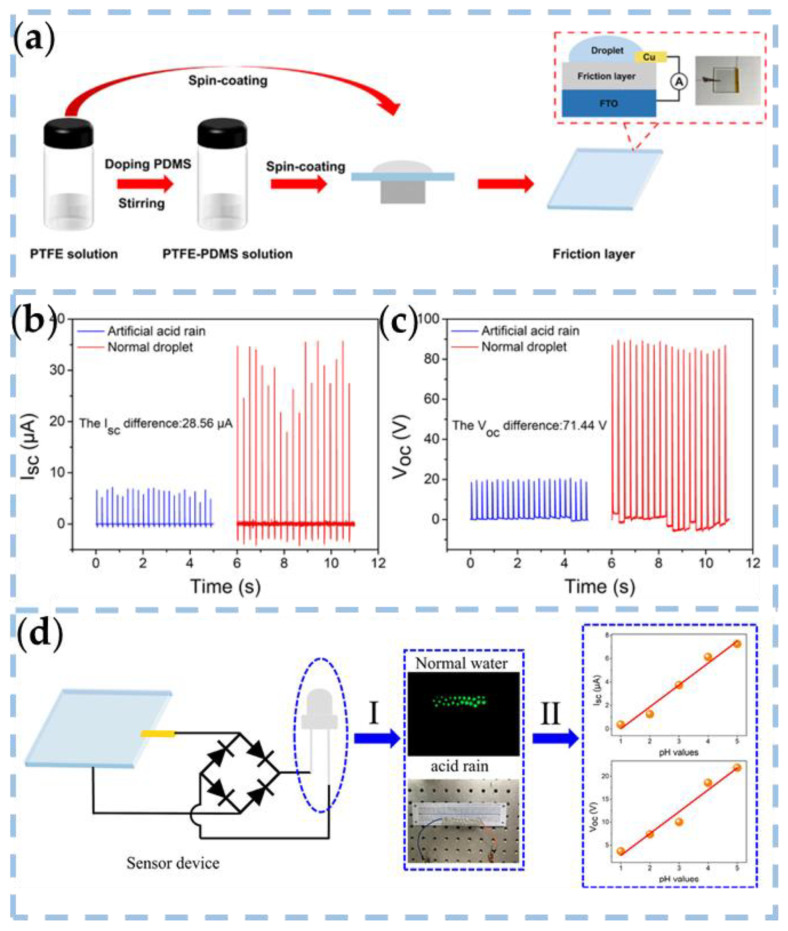
(**a**) Preparation process of the friction layer, the structure of the TENG device, and a real-time photograph of the sensor based on the D-TENG. (**b**,**c**) Differences in output performance when artificial acid rain and standard water droplets impinge on the optimized D-TENG. (**d**) Circuit diagram for detecting acid rain with the TENG sensor. Section 1 represents the visual determination of typical and acid rainwater droplets, and Section 2 illustrates the estimation of the pH value of acid rain [80]. Copyright 2021, AMER CHEMICAL SOC.

Liu et al. demonstrated a real-time acid rain sensor based on a PTFE-PDMS composite membrane nanogenerator (Figure 7a) [80]. As shown in Figure 7b,c, when standard water droplets and artificial acid rain impact the surface of the optimized PTFE–PDMS friction layer, the output signals are significantly different: 26.37μA and 69.04V, respectively. A circuit was constructed to demonstrate the real-time application of the D-TENG developed. As shown in Figure 7d, the output of the rectified TENG is used to power light-emitting diodes (LEDs) to distinguish between average rain and acid rain. The D-TENG is not only simple in structure but also requires no additional external driving force. The acidity of rainwater can be qualitatively measured in real-time. The pH value of acid rain can be roughly judged by measuring and fitting the output current or voltage curve. The experimental results show that PDMS-PTFE-based TENG has excellent pH sensitivity and linearity.

### 3.6. Self-Healing Materials

During the process of mechanical energy collection, the TENG may suffer from long-term physical impact, mechanical stiffness, and chemical corrosion, which may cause equipment failure [101,102]. The sustainability and safety of the equipment are greatly affected. In conclusion, the development of self-repairing materials can effectively improve the durability of TENG and enhance the functionalization of application scenarios. Self-healing materials mimic living organisms with self-healing properties after damage and injury. Through the reconstruction of chemical bonds, self-healing materials essentially repair losses and restore the original performance of devices. Most of the self-healing polymers studied so far are in gel form. Hydrates of hydrogels or organic gels are synthesized by fixing water or organic solvents in a self-healing polymer grid. Their structure is similar to the three-dimensional structure, which makes them complement the natural organization, with excellent adaptability and flexibility.

**Figure 8:**
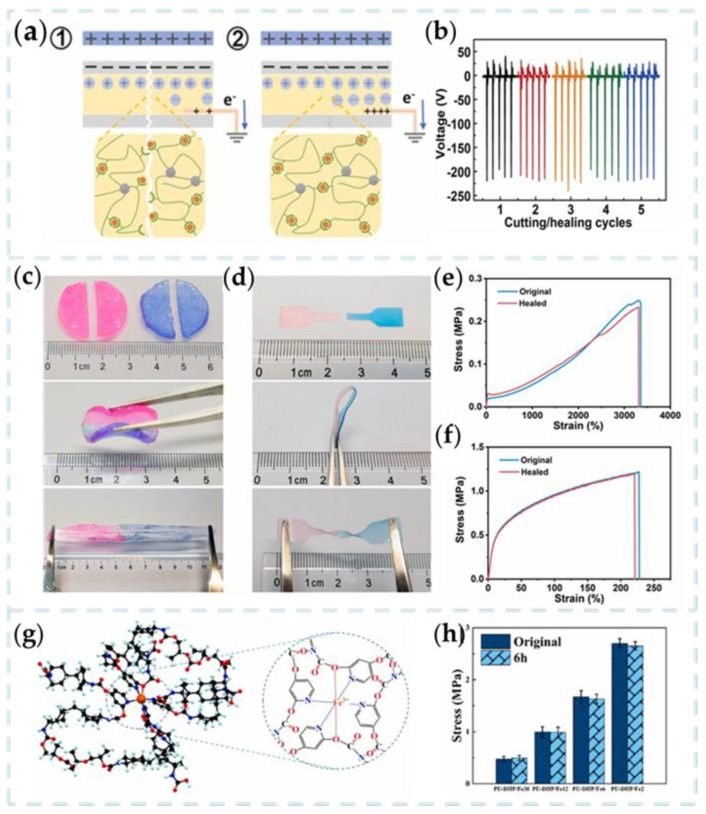
(**a**) Schematic diagram showing (1) damage to the output performance of the SI-TENG and (2) healing of the output performance of the SI-TENG. (**b**) The open-circuit voltage of the SI-TENG after different cutting/healing cycles [81]. Copyright 2022, WILEY-VCH VERLAG GMBH. Optical images of the self-healing process for (**c**) glycerol-based PAAM-Clay organohydrogel and (**d**) IU-PDMS. Stress–strain curves of (**e**) glycerol-based PAAM-Clay organohydrogel and (**f**) IU-PDMS before and after self-healing [82]. Copyright 2021, ELSEVIER. (**g**) Schematic diagram of coordination and hydrogen-bonded dual-dynamic network. (**h**) Comparison of self-healing efficiency of PU-DHP/Fe30, PU-DHP/Fe_12_, PU-DHP/Fe_6_ and PU-DHP/Fe_2_ [83]. Copyright 2021, ELSEVIER.

Li et al. proposed a transparent self-healing ion gel-based TENG (SI-TENG) [81]. The self-healing gel is synthesized by in situ polymerization of acrylic acid (AA) in 1-vinyl-3-ethyl imidazolium dicyanamide ([Emim] [OAc]) with zinc acetate dihydrate and ZnO nanoparticles (NPs). SI-TENG was prepared by assembling PAA-Zn_50_/ZnO_20_/IL_60_ ionic gel and two 3 M 9495 tape pieces into a sandwich structure. When the cut SI-TENG was rejoined, the coordination bonds on the fractured surface gradually re-formed (Figure 8a). The self-healing ability of PAA-Zn_50_/ZnO_20_/IL_60_ enables SI-TENG to maintain its original electrical output after multiple cutting/healing cycles (Figure 8b).

The self-healing characteristic provides the TENG with excellent electrical output recovery capability, which can quickly return to its original state over a wide temperature range. The novel PAAM-Clay organic hydrogel-based TENG designed by Huang et al. can operate in a wide temperature range from −30 to 80 °C [82]. As shown in Figure 8c,d, organic hydrogels, and IU-PDMS can repair themselves and form a segment that cannot be separated by mechanical stretching. Figure 8e,f shows the tensile strain curves of organic hydrogel and IU-PDMS. It can be seen that there is no significant change in mechanical properties after self-healing compared with before damage. The output properties of the new TENG are 157 V, 16μA, and 29 nC, respectively.

However, most current studies focus on preparing self-healing materials for mechanical damage, ignoring the high risk of damage caused by an electrical breakdown in TENG. Wu et al. proposed a TENG for a repairable elastomer based on a double dynamic network of hydrogen and coordination bonds [83]. Figure 8g shows a schematic diagram of the two-dynamic network of coordination and hydrogen bonds, giving the unique material properties. After the breakdown of multiple electric fields, the mechanical self-healing efficiency of TENG is still 96%, and the electrical self-healing efficiency is almost 100% (Figure 8h). It provides an excellent idea for TENG to avoid mechanical or electrical breakdown damage.

### 3.7. Shape-Memory Polymers Materials

Shape-memory polymers (SMPs) are a class of responsive materials that can change their temporary shape and recover their original shape intact in response to external stimuli. This process is due to the dissociation–association process that occurs within the dynamic interaction of the polymer [103,104]. Due to their dielectric properties, wide electron affinity range, flexibility, and barrier properties, SMPs can be used as contact materials for TENG, providing considerable benefits to the device [105]. For instance, polyurethane SMPs fabricated through photolithography have been used to prepare pyramid-patterned TENGs, which can improve the device’s lifespan [106]. This approach may represent a promising direction to enhance the durability and functional diversification of TENG devices.

**Figure 9:**
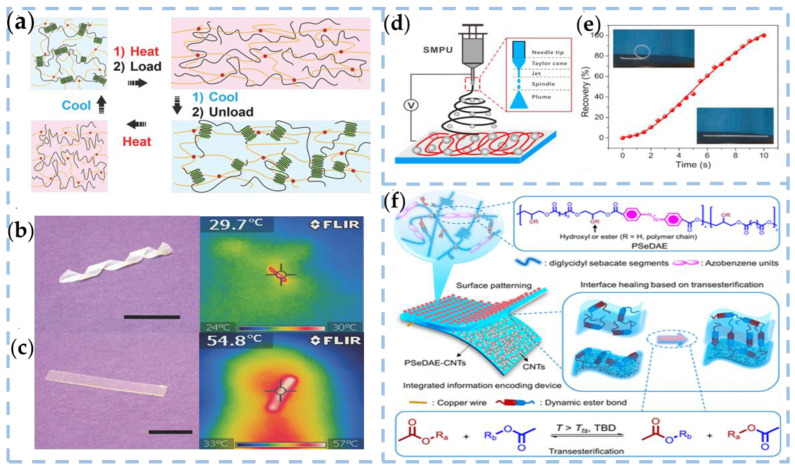
(**a**) Schematics showing the mechanism of shape-memory behavior. (**b**) Sample with temporary spring shape and (**c**) after reverting to the original condition along with the corresponding thermal images [84]. Copyright 2018, WILEY-VCH VERLAG GMBH. (**d**) Schematic illustration of the electrospinning process of SMPU microarchitectures. (**e**) The recovery ratio of a representative MFs mat as a function of the heating time at 75 °C. Insets are photographs of a mat ribbon in the initial circular shape and the completely recovered form [85]. Copyright 2019, ELSEVIER. (**f**) Schematic illustrations of the PSeDAE and resultant IED with an integrated structure [86]. Copyright 2022, AMER CHEMICAL SOC.

Liu et al. reported an SMP-based TENG (STENG) to capture biomechanical energy and detect biomechanical motion [84]. The SMP was synthesized by incorporating a semicrystalline thermoplastic polymer in a chemically cross-linked elastomer. The mechanism of shape-memory behavior is shown in Figure 9a. The shape-memory properties of semi-IPN elastomers are qualitatively demonstrated using thin films. Flat films deform into different shapes (spring-like in Figure 9b) with higher temperatures. Shape recovery is triggered by changing the room temperature (Figure 9c). By utilizing the macroscopic shape-memory ability of SMP, a wrist splint capable of shape adaptation for mechanic sensation was developed using a blend of photocurable elastomer and polycaprolactone SMP film. Additionally, this SMP film was utilized as an energy harvester for body motion. Although this is only a rough model, it has a simple manufacturing process and broad feasibility.

In addition, SMPs also can be implemented under non-thermal stimuli such as water/humidity, photoactivity, electroactivity, and magnetic activity. As shown in Figure 9d, Xiong et al. broke through the research gap of water-activated microarchitecture shape-memory TENG with water energy harvesting and water temperature sensing capabilities. They proposed a multifunctional TENGs electro-spun microstructure pad with self-recovery capability [85]. Three scalable microfibers (MF), microspheres (MS), and microsphere-nanofiber (MSNF) pads were fabricated by electrospinning. The types of micro-structured shape-memory TENG provide triboelectric sound output (~150–320 V, ~2.5–4 μA cm^−2^). The shape-memory capability of the display can be adapted to a variety of temporary shapes, and the recovery rate of 100% can be achieved quickly in 10 s (Figure 9e). The results show that electrospinning is an effective method for constructing layered microstructures without losing shape memory.

In a dynamic interaction, SMPs store only one temporal shape per shape-memory loop. Therefore, the dual shape-memory effects of heat-sensitivity, light-sensitivity, and chemical sensitivity can be achieved by using reversible dynamic bonds. Further, SMPs with triple or multiple shape-memory effects are created using noninterfering reversible interactions of two or more responses to different stimuli and combined into a system. Xuan et al. synthesized a high-permittivity poly(sebacoyl diglyceride-co-4,4′-azo dibenzoyl diglyceride) elastomer (PSeDAE) with light-to-thermal conversion and triple-shape-memory [86]. PSeDAE as a triboelectric layer constructs an Information Encoding Device (IED) based on TENG (Figure 9f). TENG can produce tri-shape-memory effects, inducing both microscopic and macroscopic functions. The micro-scale shape-memory effect enhances the electrical memory properties of IED, enabling program editing capabilities. Meanwhile, it enables the on-demand transformation of wearable applications through macro-scale shape memory.

### 3.8. Multiple-Stimulus-Responsive Materials

The TENG based on stimulus-response materials is a simple and efficient device that can be accurately controlled and easily prepared. However, in real-world environments, the stimulus sources are diverse, and the application scenarios are complex and variable. Therefore, to meet the diverse requirements of such environments and to enhance the intelligent response of TENG output, a single stimulus-response material is often insufficient. To address this issue, research is currently focusing on developing updated response conditions and diversified response methods. One promising approach is the use of multi-stimulus response smart materials, which incorporate multiple functional stimulus-response groups into polymer materials. Through precise molecular structure design and effective preparation methods, these materials can achieve multiple reaction properties and explore the practicality of smart response TENG functions.

**Figure 10:**
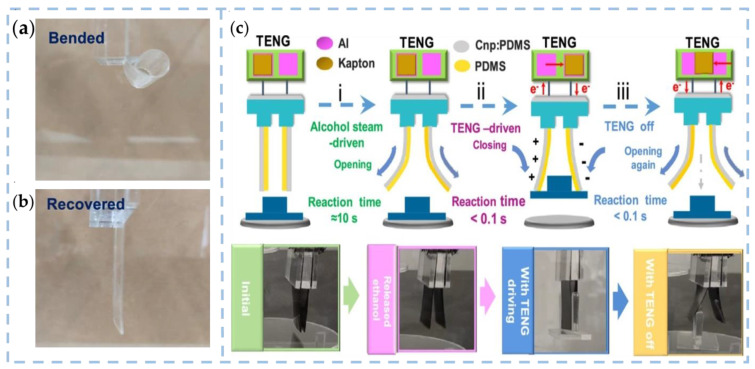
(**a**,**b**) Curling and recovery process of the PDMS film [65]. Copyright 2019, AMER CHEMICAL SOC. (**c**) Working principle and three working processes of the dual-stimulus flexible gripper based on TENG and vapor induction: (i) ethanol vapor drives the gripper to open, (ii) TENG drives the gripper to hold the object, (iii) TENG reopens the gripper and unloads the object [107]. Copyright 2022, ELSEVIER.

Furthermore, certain polymers exhibit sensitivity to humidity as well as other stimuli, allowing for a diverse range of design functionalities. Li et al. have demonstrated the design of two flexible actuators and grippers that can respond to dual stimuli of ethanol steam and electrostatic power, as illustrated in Figure 10a,b. These devices are capable of completing a series of tasks such as loading, driving, transporting, and unloading objects without requiring an external power supply [65]. They can complete a series of motion tasks such as loading, driving, transportation and unloading objects without an external power supply. Xiang et al. proposed a self-powered triboelectric mechanical luminescent electronic skin (STMES) capable of detecting and discriminating between multiple stimuli [107]. An electronic skin sensor with an electro-optical dual-signal sensing mode is designed by combining the TENG and mechanical luminescence (ML). STMES operates under the synergy of TENG and ML. As shown in Figure 10c, the electrical signal senses normal pressure or bending stress, while turning or transverse tensioning stimulates the optical signal of the mechanical luminescence spacer. Different responses in the form of electrical/optical signals distinguish mechanical stimuli.

TENG provides a straightforward and effective means to drive many electrically responsive materials and devices, leveraging its unique output characteristics. The output voltage and transmitted charge of the TENG can be precisely controlled by selecting specific operating parameters, enabling the drive and operation of these systems. To effectively navigate and control a range of TENG-based functional systems, it is crucial to maintain high output voltage levels.

## 4. Applications of TENGs That Are Coupled with Smart Materials

### 4.1. Magnetic Field Detection

The field of magnetic sensing has greatly benefited from the development of various types of magnetic sensors, such as biological magnetic sensors, GMI magnetic sensors, magnetic harmonic sensors, and magnetometers. However, these commonly used sensors have limitations that need to be addressed, such as complex circuits, high cost, and nonlinearity in demodulation, which can hinder their widespread use in practical applications. To overcome these challenges, researchers have focused on developing magnetically responsive materials that can be used to monitor magnetic fields with high accuracy and sensitivity. The development of such materials has led to the creation of innovative magnetic sensors that can provide reliable and precise measurements of magnetic fields in a wide range of settings, from scientific research to industrial applications. As early as 2012, Wang et al. proposed a magnetic field monitoring system based on the triboelectric nanogenerator (TENG), which combines polydimethylsiloxane (PDMS) and a metal (Fe) disc for magnetic field sensing [108]. PDMS serves as the dielectric that generates friction charges, while a metal (Fe) disc is connected to the other end of the TENG as the sensitive unit for the magnetic field, with a solenoid coil placed underneath the disc. When current flows through the solenoid coil, the Fe disc is attracted by the electromagnetic force, causing the TENG to bend downward. The resulting deformation and deformation rate lead to the electrical output signal of the TENG.

**Figure 11:**
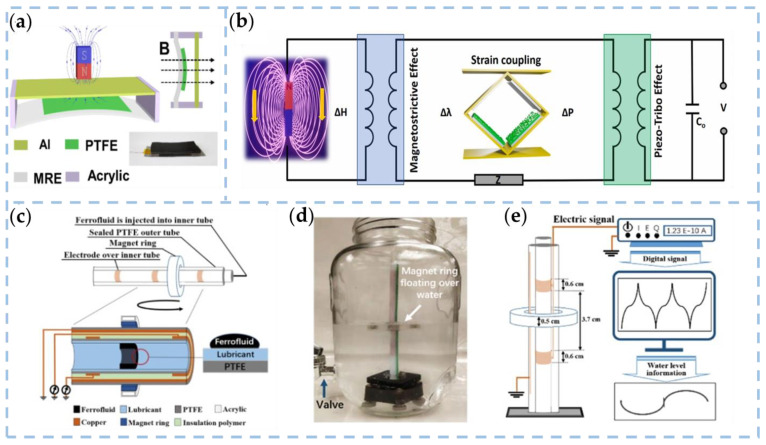
(**a**) The structural design of the MRE-based TENG device [109]. Copyright 2021, MDPI. (**b**) Mechanism of mechano-magnetic and magneto-electric coupling [110]. Copyright 2021, ELSEVIER. (**c**) The schematic of magnetic field assisted non-contact LLI-TENG. (**d**,**e**) The photograph of LLI-TENG for the water level sensor and the schematic of the detection process [111]. Copyright 2020, ELSEVIER.

Wan et al. prepared MRE films by embedding MFD in styrene-butene-styrene (SEBS) and designed the pointer TENG structure [109]. MRE is a magnetically sensitive smart composite that external magnetic fields can rapidly and reversibly control. As shown in Figure 11a, a vertical magnetic field is applied to the TENG, and the MRE film deforms under the magnetic field, driving the PTFE close to the Al electrode. The MRE-based TENG has excellent output performance with an open circuit voltage of 16 V, a short circuit current of 0.18 μA, and a maximum output power of 1.3 μW at a voltage of 80 M Ω. The developed magnetic sensor based on the MRE film has a fast response capability of 80 ms and an ultra-high sensitivity of 31.6 mV/mT in the magnetic field range of 35 to 60 mT. With the help of a programmable platform, the initial vectorization capability and unique self-powered capability are realized for real-time magnetic field monitoring, which further promotes the application of tribometer technology in sensing. Sugato Hajra et al. used triboelectric and piezoelectric effects to create a hybrid nanogenerator device based on multiferroic materials (MF-HG) (Figure 11b) [110]. The rare earth regular ferrite ErFeO_3_ is doped into the ferroelectric BaTiO_3_ without disturbing its lattice. It was experimentally demonstrated that 3% ωt. EFO in BTO and 10% ωt. BTEFO in PDMS would produce the best electrical output (260 V and 3 µA). The designed multi-cell MF-HG can be used as a functional sensor to sense the external magnetic field. The research and analysis conducted in this study show that the MF-HG device has the potential to serve as a promising candidate for self-powered electromagnetic sensors. Moreover, MF-HG may have practical applications in magnetic sensing, mineral exploration, defense, and environmental monitoring in the near future.

In the actual application of the TENG, in addition to its mechanical losses, the friction layer is easily stained by biological and chemical stains from the external environment. Therefore, stains on the interface will reduce the output capacity of the TENG and bring bias to the TENG sensor. Wang et al. proposed a novel magnetic field-assisted non-contact liquid–liquid interface TENG (LLI-TENG) to solve the above problems of traditional TENG in self-powered sensing applications [111]. As shown in Figure 11c, to achieve non-contact behavior, ferrofluid is injected into a sealed polytetrafluoroethylene (PTFE) tube coated with a copper (Cu) electrode. The ferrofluid flows smoothly through the line under the action of the external ring. Electrons are transferred back and forth between the copper electrode and the ground. In order to construct the liquid–liquid triboelectric interface and promote the sliding behavior of the ferrofluid at the interface, an oil-perfluorinated polyether (PFPE) was introduced into the interface between the ferrofluid and the PTFE tube. This method expands the range of the linear relationship between the friction current peaks and increases the maximum detection velocity value of LLI-TENG from 0.1 cm/s to 5 cm/s. The study highlights the benefits of using a non-contact liquid–liquid interface TENG as a self-powered water level sensor. Notably, the TENG performs exceptionally well in simulating polluted water environments (Figure 11d,e), indicating its strong immunity to harsh conditions.

### 4.2. Body Condition Monitoring

Precise monitoring of body movements is crucial for health assessment and early medical diagnosis, particularly for the elderly and injured individuals. The camera-based approach is considered one of the most efficient ways to capture gait movement. Nonetheless, these devices have limited sensing and deformation abilities, as well as limited durability, which renders them inadequate for motion monitoring applications. As a result, the development of novel technologies that offer wearable, user-friendly, and accurate motion detection features remains a significant challenge.

**Figure 12:**
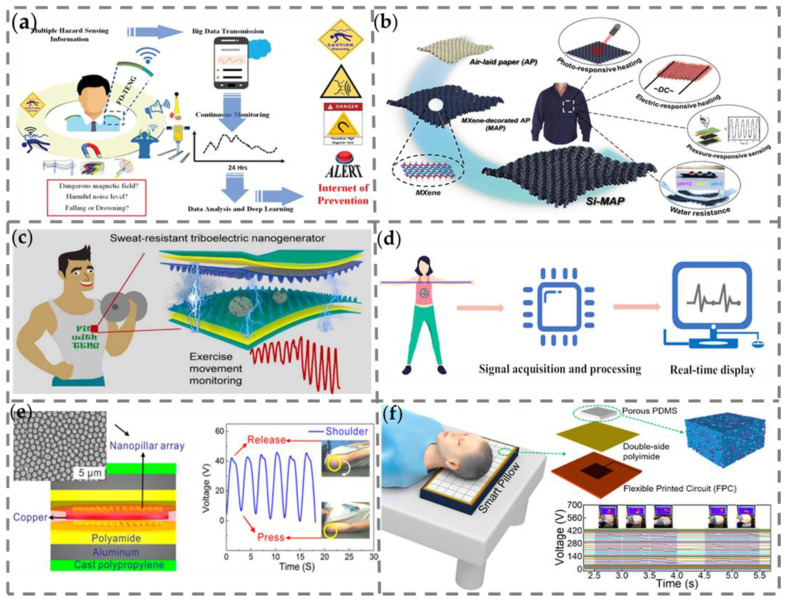
(**a**) Hazard preventive wearable platform. Different forms of mechanical force are typically obtained by the human body or induced externally by magnetic or acoustic fields [112]. Copyright 2019, WILEY-VCH VERLAG GMBH (**b**) Schematic illustration of the preparation process of the hydrophobic MXene-decorated fabrics [113]. Copyright 2020, AMER CHEMICAL SOC. (**c**) Schematic structure of the BSRW-TENG [114]. Copyright 2022, ELSEVIER. (**d**) Real-time demonstration process in the actual application of the self-power smart elastic belt system [115]. Copyright 2021, AMER CHEMICAL SOC. (**e**) Application of the TES for sleep–body monitoring [116]. Copyright 2016, AMER CHEMICAL SOC. (**f**) Application of the FB-TENG array as a smart pillow in monitoring head movement [117]. Copyright 2022, AMER CHEMICAL SOC.

Ahmed et al. have proposed a multimodal ferrofluid tribological nanogenerator (FO-TENG) with significant stretchability and diversified sensing capabilities and excellent verifiability [112]. The smart sensing platform developed through FO-TENG can facilitate danger signals by activation/stimulation of different mechanical stimuli, including endogenous forces such as compression, tension, or vibration (body movement), or even remotely trigger the system by exogenous forces such as magnetic fields, sound waves, or radio (Figure 12a). It is worth noting that MXene is a material with high thermal conductivity and high photothermal conversion behavior so it can be easily applied in both photo-responsive and electrical-responsive applications. Moreover, MXene is compatible with textile substrates without reducing its conductivity, which is due to its abundance of end groups. Ma et al. designed a wearable, hydrophobic MXene-decorated airstream mesh paper (Si-MAP) through a simple dip-coating strategy [113]. The manufacturing process is shown in Figure 12b. The response time and recovery time of the pressure sensor based on Si-MAP are ~40 and ~30 ms, respectively. It is expected to be used as a wearable electronic textile in human motion monitoring.

Currently, exercise movement monitoring and analysis can effectively regulate incorrect movements and thus effectively ease the suboptimal health status (SHS) body. Moreover, dynamic analysis of athletes’ exercise movements can enable them to discover tiny imperfections in their training and thus improve their performances. Li et al. proposed a biologically responsive sweat-proof wearable triboelectric nanogenerator (BSRW-TENG) for motion monitoring during exercise [114]. As shown in Figure 12c, the BSRW-TENG consists of a superhydrophobic and self-cleaning friction-charged layer (elastic resin and polydimethylsiloxane (PDMS)). The hierarchical micro/nanostructure replicated by lotus leaves not only achieves twice the output increase of BSRW-TENG but also provides BSRW-TENG resistance with excellent pollution and humidity, constituting sweat resistance. The BSRW-TENG has great potential for low-cost monitoring of individual sports and analysis of athlete training. In the study of wearable electronic devices, most current conductors possess poor electromechanical performance and adhesion of fillers on elastomers due to uneven mixing, thus leading to insufficient compatibility between conductive fillers and adjacent elastic networks. Sheng et al. have synthesized the hydrogel with zinc (Zn) in a polymer strategy with sodium alginate (SA) and poly(acrylic-acrylamide) (PAA) [115], and they have introduced an ultra-stretchable TENG based on a tensile ionic conductor hydrogel. Experiments have shown that Zn hydrogel TENG exhibited good electrical output performance even under high-tensile states. In addition, a TENG self-powered elastic training band sensor based on conductive hydrogel was developed for monitoring data in stretching exercises (Figure 12d). The proposed TENG may have potential applications in fields of medical monitoring and rehabilitation, electronic skins, self-powered sensors, and human–computer interaction in the future.

Nowadays, sleep disorders are major health problems affecting many people. At the same time, health monitoring products have gradually entered daily life with the functions of blood pressure monitoring. Traditional sleep monitoring products might be affected by poor wearing comfort, low detection sensitivity, radiation, and external power supply. Therefore, it is imperative to develop a sleep monitoring product with a simple structure, low cost, self-powered supply, noninvasive usage, comfortable experience, and high sensitivity. Song et al. have introduced a sensitive TENG based on an aluminum–plastic laminated film (APLF) serving as a self-powered device [116]. The sensitive and flexible TENG based on nanopillar arrays on APLF is easily packaged and used as a self-powered TES for monitoring the overturning movement of a shoulder and a leg during sleeping (Figure 12e). As a sleep monitoring product, the most critical features are comfort and convenience. Kou et al. have developed a pressure-sensitive, noninvasive, breathable, and comfortable smart pillow [117]. The smart pillow is based on a flexible and breathable triboelectric nanogenerator (FB-TENG) pressure sensor array for head movement monitoring and the falling-out-of-bed alarm function (Figure 12f). The FB-TENG is assembled based on porous poly(dimethylsiloxane) (PDMS) containing a fluorinated ethylene propylene (FEP) powder produced by the sacrificial template method. Based on the FB-TENG, an 8 × 8 sensor array was integrated. This work is expected to provide another possibility for the field of human–machine interactive sleep behavior monitoring.

### 4.3. Soft Robots

Soft robots inspired by living organisms have shown tremendous promise. Soft robots can provide safer and more robust human–computer interaction than their rigid counterparts. Their deformability and drive performance can adapt to complex environments and relatively free movement. Stimulus-responsive soft robots, as a new type of intelligent system used for multifunctional actuators or sensors, have attracted much attention. However, it is not easy to develop sensors that can work on soft robots. They will face problems such as complex device structures, poor stretchability, poor performance, and incompatible moduli. The self-feedback soft robots based on TENG technology have the advantages of good design flexibility, simple installation, and light weight, which opens up new avenues for the development of self-assembling soft robots.

**Figure 13:**
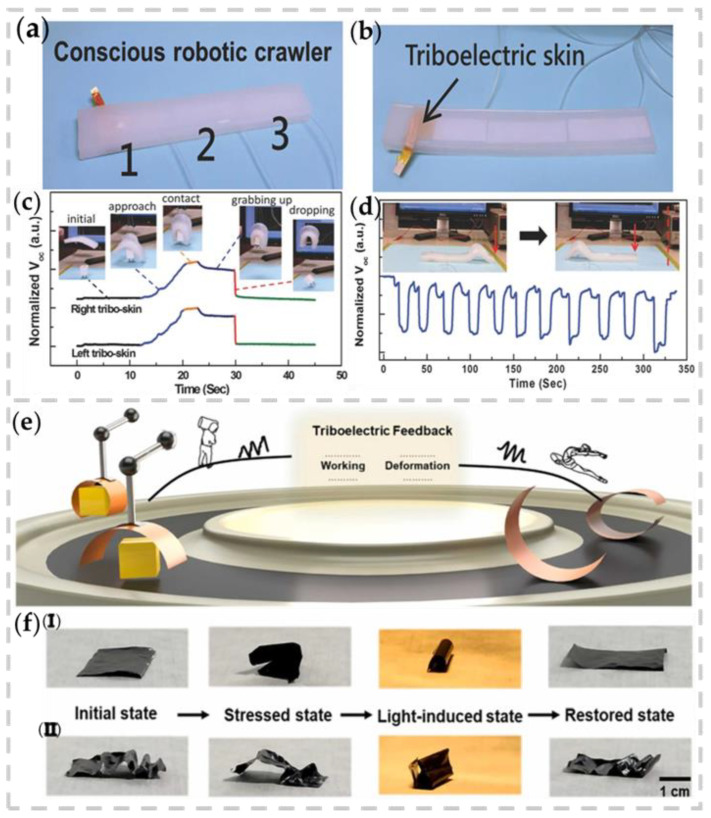
(**a**) Image of conscious robotic crawler(1, 2, and 3 are three signal ports). (**b**) The appearance of the abdomen of a conscious robotic crawler and tribo-skin. (**c**) Tribo-skins enable soft robotic gripping to finger to perceive working states actively. Real-time outputs of right and left tribe-skins when conscious gripper grabbed and dropped an object. (**d**) Real-time results of conscious crawler during crawling ≈15 cm distance [118]. Copyright 2018, WILEY-VCH VERLAG GMBH. (**e**) Schematic of the contact feedback of the PCPA. (**f**) Photographs of PCPA demonstrating the shape-memory diversity [119]. Copyright 2022, ELSEVIER.

Lai et al. first demonstrated a variety of soft robots enabled by self-powered, highly stretchable, and highly sensitive triboelectric skin [118]. The triboelectric effect can actively perceive and respond to external stimuli and internal motion. First, as shown in Figure 13a,b, the pressure-sensitive friction skin is embedded into the abdomen of the crawling robot and its ability is tested. Figure 13d shows the real-time output after the robot has moved a distance of ≈15 cm, indicating the reliability of the rubbed skin. Figure 13c illustrates a perceptive soft grip. The actively generated signals show that the gripper can sense different movements of grasping objects and is aware of items falling, indicating that they can be applied to the monitoring and feedback control of industrial robots.

Jin et al. proposed a kind of contact feedback thin film soft actuator based on photothermal effect and friction nanogenerator: PET-carbon Black ink-PDMS actuator (PCPA) (Figure 13e) [119]. It is noteworthy that PCPA can achieve mechanical memory without shape-memory materials. It can withstand mechanical stress in any direction. As shown in Figure 13f(i–ii), it bends under light stimulation and returns to its initial state after turning off the light, demonstrating the shape-memory diversity of the soft actuator.

### 4.4. Biomedicine

The increasing demand for health monitoring and personalized medicine has driven the design and development of TENG-based wearable biosensors. Biomechanical energy, provided by human movement, can be collected by TENGs to generate high-quality electrical signals, enabling real-time measurement of physical parameters such as pulse, heart rate, temperature, and blood pressure [120]. The support of internet- and wireless network technology allows for sustainable and personalized physiological monitoring of the human body, where TENGs can quantify various biochemical markers noninvasively and provide personalized physiological parameters. TENG-based therapeutic tools have been developed for clinical treatment, showing excellent performance in tissue repair, cell proliferation, and nerve prostheses. TENG-based self-powered systems provide the possibility to solve the limitations of traditional biomedical equipment by operating in a self-powered mode, accurately identifying the health status of the body, and treating chronic diseases with a flexible plan through continuous feedback of data [121]. Advancements in materials science, mechanical engineering, and wireless communication technologies have optimized the intelligence and mobility of TENG-based self-powered systems for providing customized medical services and personalized medical solutions in various applications, such as drug delivery, wound healing, nerve regulation, and respiratory sensing.

**Figure 14:**
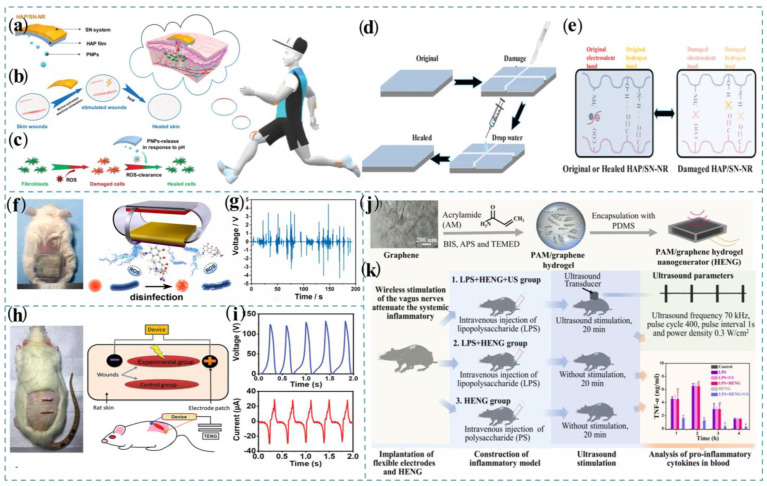
(**a**) HAP/SN-NR comprises utilized film and a self-powered nanogenerator (SN). (**b**) The work process of the SN system under motion-activated electrical stimulation to make skin convalescent. (**c**) The ROS-clearance of HAP film with ROS and pH dual response to rehabilitate damaged human dermal fibroblasts. (**d**) The diagrammatic sketch of the self-healing process. (**e**) The self-healing mechanism of HAP film [122]. Copyright 2021, WILEY-VCH VERLAG GMBH. (**f**) Photograph of the mouse wearing the MSETENG patch on infected wounds in the back and the proposed mechanism for promoting infected wound healing. (**g**) Output voltages of the MSETENG patch induced by the mouse’s motion [68]. Copyright 2021, ELSEVIER. (**h**) A schematic system set up by B-TENG to induce ES for bruise therapy. (**i**) The condition of output voltage and current of B-TENG for ES [123]. Copyright 2022, WILEY. (**j**) The fabrication processes of HENG. (**k**) Schematic representation of experimental protocols of the ultrasound-driven electrical stimulation of vagus nerves for attenuating the endotoxin-induced systemic inflammation [124]. Copyright 2021, ELSEVIER.

Yuan et al. designed a portable self-powered stimulated nanocomposite repair (NR) based on a frictional nanogenerator and an intelligent nanoparticle delivery system [122]. The cooperation of the film prepared by layer-by-layer self-assembling 2-hydroxypropyl trimethyl ammonium chloride chitosan (HTCC), alginate (ALG), and poly-dopamine/Fe^3+^ nanoparticles (PFNs), with a self-powered nanogenerator (SN) driven by moving into a nanocomposite repairer (HAP/SN-NR) is conducted (Figure 14a,b). The system can provide a strong electric field and stimulate epidermal regeneration, which is conducive to angiogenesis and rapid active skin healing. More importantly, when the wound state changes from neutral to slightly acidic, polydopamine nanoparticles (PNP) from HAP films can be released to the injury site in response to maintaining cell health (Figure 14c). The damaged area of the film deforms after contact with water, which is specifically manifested as the beginning of the rapid expansion. In addition to this, the mobility of PNPs and the movement of polyelectrolyte ALG and HTCC chains are enhanced, leading to an enhanced aggregation affinity at the site of membrane damage. It can be seen that water molecules play an important role in the self-healing mechanism of HAP/SN-NR films. Figure 14d,e show the self-repair process and self-healing mechanism of HAP films. Furthermore, electrical stimulation (ES) has recently attracted widespread attention in wound treatment due to its superiorities in aspects of promoting cell migration, collagen deposition, angiogenesis, etc. Du et al. presented an integrated and rectifier-free TENG patch with surface-engineered electrodes for the drug loading/release and locally producing ES to accelerate the healing of infected wounds [68]. The results of in vitro and in vivo tests show that the skin patch can fit the wounds, and simultaneously produce ES and release minocycline in a controlled manner for inhibiting the wound bacteria (~ 96.7%, S. aureus) and benefiting skin tissue repair (Figure 14f). Upon in vivo application, the MSETENG generated AC voltage (0.5–4.5 V) and current (5–40 nA) induced by the mouse’s motions (Figure 14g). This work opens a new route for combating wound bacterial infection and facilitating skin regeneration through a convenient and personalized solution.

Compared to incisional wounds, bruises and pressure sores caused by impact or pressure are also injuries that need to be paid more attention to, especially for long-term bedridden patients and people with disabilities who cannot move by themselves. Chen et al. reported a facile, fabric-compatible, and flexible B-TENG based on the borophene/ecoflex nanocomposite [123]. The borophene/ecoflex nanocomposite possesses high surface charge density, cost-effectiveness, large scalability, flexibility, and durability. the animal model test was employed to confirm the feasibility of using B-TENG in real wound-healing applications (Figure 14h). B-TENG served as a self-powered therapeutic unit to treat the as-designed wound, where the wound recovery was statistically evaluated by the animal model. The output performance of B-TENG was fixed at 30 µA current and 125 V voltage within the measurement, respectively, as shown in Figure 14i. It is expected that the as-designed B-TENG will not only manifest the progress of self-powered healthcare sensors but also benefit future technological applications from assistive technology to wearable therapeutics.

Exploring micro or soft implantable bioelectronic installations with wirelessly powered or self-powered technology is a rapidly growing field of research. Ping et al. presented a battery-free vagus nerve stimulator that uses an implantable hydrogel nanogenerator (HENG) for power [124]. The generator was developed based on a polyacrylamide/graphene conductive hydrogel (Figure 14j). Since the ultrasonically induced vibration of the compressible electric double layer is at the conducting hydrogel/electrolyte interface, HENG can be remotely driven to generate induced current using programmable ultrasonic pulses. To test the ability of PAM/graphene HENG to stimulate the vagus nerve in vivo in anti-inflammatory therapy, systemic inflammation was induced in experimental mice by intravenous injection of lipopolysaccharide (LPS) at a median lethal dose (10 mg/kg) (Figure 14k). After implanting HENG, it was excited using ultrasound in real time with programmable ultrasound pulses to generate AC pulses for vagus nerve stimulation. After electrical stimulation, the inhibitory effect of vagus nerve stimulation on proinflammatory cytokines was evaluated by blood analysis of rats.

## 5. Summary and Perspectives

Smart materials that are combined with TENG may present superior sensing and responsive capabilities for broad development prospects about artificial intelligence, the Internet of Things, smart healthcare, etc. In this review, we briefly summarize the basic concept and the recent progress of TNEG-based self-powered systems that utilize the advantages of smart materials. Although the functions of these smart materials and the TENG-based systems may differ in structure, working principle, and integration strategy, the integrated systems all take advantage of the smart responsive properties, either in enhancing energy conversion efficiency or obtaining sensitive features. As a result, TENG-based materials reach the same end by different means by combining self-healing materials, and shape-memory materials, for wider applications in biomedicine, smart wearables, and soft robotics. Nevertheless, to stride the span from laboratory scale to practical application, the prospective development of TENGs that are based on smart materials might be strengthened in the following aspects:

1. Enhance the synergistic effect of electrical conductivity and mechanical properties of smart materials.

To open up new avenues for advanced applications of multi-functional systems, it is crucial to further investigate the fundamental characteristics of smart materials, such as compatibility and flexibility. However, achieving the desired performance of conductivity and mechanical behavior poses a challenge due to their contradictory recovery efficiencies. Hence, future studies should focus on developing a better understanding of the synergistic effect of high conductivity and mechanical properties. This requires multi-scale theoretical simulations and modeling to elucidate the molecular-level repair mechanisms. By gaining insights into the nanoscale to micron scale, innovative concepts, theories, and scalable manufacturing techniques can be designed to fabricate new smart materials.

2. Develop multiple functions based on the TENG features.

The potential of traditional response materials and devices can be further enhanced by leveraging the universal applicability and diversified structural design of triboelectric nanogenerators. This technology can facilitate the miniaturization of large operating systems and provide self-protection capability for high-voltage devices, which traditional power sources cannot achieve. Additionally, TENG-based systems can simplify feedback/control circuits and enable instant human-computer interaction.

3. Explore various materials and devices coupled to the TENG.

To develop an efficient and intelligent system, the response materials device applied to TENG should satisfy the following basic conditions. Firstly, in order to match the characteristics of high output voltage and low current of the TENG, the picked materials and devices are preferably voltage-adjustable systems. Secondly, the picked materials and devices coupled to the TENG must have excellent insulation properties (capacitor pieces) to diminish leakage current and benefit to keep the high voltage of the nanogenerator. Thirdly, the target system must work with low power consumption due to less charge transfer during the motion period of the electrodes. Ultimately, the distinctive capabilities of the target materials and equipment should help to broaden the spectrum of applications for TENG-based self-power systems.

4. Reliability of TENG-based self-powered systems.

In the current research stage of intelligent sensor systems based on TENG, most researchers focus on achieving sensing capability and the sensing accuracy of TENG has not received adequate attention. The sensing accuracy of TENG can be improved by modifying materials, designing structures, and managing power to enhance its electrical performance. In practice, many external environmental factors can damage the steadiness—humidity, for example. This will be a significant obstacle to TENG-based development promotion. One effective solution is to enhance the hydrophobicity and self-cleaning of the friction layer by modifying its surface with hydrophobic chemical groups or creating surface nanostructures. Another promising approach is to develop advanced packaging techniques that can shield TENG devices from moisture penetration while maintaining their electrical performance.

## Figures and Tables

**Table 1:** Comparison of synthesis methods, properties and applications of TENGs combined with different types of smart materials.

Type	Material	Method of Synthesis	Performance	Application	References
Magnetic responsive materials	γ-Fe_2_O_3_, PVDF	Spin coating, Solution casting	0.8 µA, 90 V	Scavenging energy from rotary pump vibrations.	[69]
	Al/Ni fabric, PDMS	Solidified stripping	30 µA, 205 V	Harvesting wind energy.	[70]
	NdFeB magnets	Solution casting	1.17 mA·cm^–2^	Harvesting wind energy.	[71]
Optical responsive materials	ZnO-NPs, PVDF	Solution casting	28 V	Monitoring of movement.	[72]
	PEDOT-MeOH	Sol-gel method, Spin coating	-	Self-powered smart windows	[73]
Hygro-responsive materials	Chitosan-glycerol film	Coating method	130 V	Sweat sensor, Monitoring of movement.	[74]
	PFSA ionomer	Solution casting, Spin coating	-	Self-powered steam sensor	[75]
	SnS_2_	Chemical vapor deposition	7.6 µA	Humidity sensor	[76]
Temperature-responsive material	PCL, fluorinated alumina	Solid–liquid mixing	85 nA, 6 V	Wetting monitoring and temperature sensing	[77]
	CNFs, AgNWs, PA, BP	Vacuum filtration	116 V, 3.8μA, 42 nC, 33.4 μm^−2^	Fire warning, Temperature sensing	[78]
	NIPAM, MMA	Free radical polymerization and spin-coating.	242.72 nA, 0.72 V, 10.78 nC	A stable warning system for real-time liquid temperature monitoring	[79]
pH-responsive materials	PTFE, PDMS	Spin coating	26.37 μA, 69.04 V	Acid rain sensor	[80]
Self-Healing materials	acrylic acid,1-vinyl-3-ethyl imidazolium dicyandiamide, ZnO NPs	Solution casting	3.15 W·m^−2^	Monitoring of movement	[81]
	AAM, KPS, glycerol, NH_2_-PDMS-NH_2_	Solidified stripping	157 V, 16 µA, 29 nC, 710 mW·m^−2^.	Monitoring of movement	[82]
	PU	Solution casting	180 V, 1.3 μA	Monitoring of movement	[83]
Shape-memory polymers	PCL, n-butyl acrylate	Solidified stripping	-	Smart wrist splint with an alarm system.	[84]
	Polyurethane	Electrospinning	150–320 V, 2.5–4 μA·cm^−2^	Self-powered water temperature sensor.	[85]
	PSeDAE	Cross-linking, Laser engraving	44.5 V, 1 μA, 17.5 nC	Improvised explosive device.	[86]

## Data Availability

Data are available on request from the authors.

## References

[1] Zheng Q., Xu C., Jiang Z., Zhu M., Chen C., Fu F. (2021). Smart Actuators Based on External Stimulus Response. Front. Chem..

[2] Wei J., Li R., Li L., Wang W., Chen T. (2022). Touch-Responsive Hydrogel for Biomimetic Flytrap-Like Soft Actuator. Nano-Micro Lett..

[3] Long F., Cheng Y., Ren Y., Wang J., Li Z., Sun A., Xu G. (2022). Latest Advances in Development of Smart Phase Change Material for Soft Actuators. Adv. Eng. Mater..

[4] Jingcheng L., Reddy V.S., Jayathilaka W.A.D.M., Chinnappan A., Ramakrishna S., Ghosh R. (2021). Intelligent Polymers, Fibers and Applications. Polymers.

[5] Wang W., Li P.-F., Xie R., Ju X.-J., Liu Z., Chu L.-Y. (2022). Designable Micro-/Nano-Structured Smart Polymeric Materials. Adv. Mater..

[6] Haq M.A., Su Y.L., Wang D.J. (2017). Mechanical properties of PNIPAM based hydrogels: A review. Mater. Sci. Eng. C-Mater. Biol. Appl..

[7] Das S., Ngashangva L., Goswami P. (2021). Carbon Dots: An Emerging Smart Material for Analytical Applications. Micromachines.

[8] Hajra S., Sahu M., Sahu R., Padhan A.M., Alagarsamy P., Kim H.-G., Lee H., Oh S., Yamauchi Y., Kim H.J. (2022). Significant effect of synthesis methodologies of metal-organic frameworks upon the additively manufactured dual-mode triboelectric nanogenerator towards self-powered applications. Nano Energy.

[9] Kim Y.-J., Matsunaga Y.T. (2017). Thermo-responsive polymers and their application as smart biomaterials. J. Mater. Chem. B.

[10] Chen Z., Liu J., Chen Y., Zheng X., Liu H., Li H. (2021). Multiple-Stimuli-Responsive and Cellulose Conductive Ionic Hydrogel for Smart Wearable Devices and Thermal Actuators. Acs Appl. Mater. Interfaces.

[11] Niu Y., Li S., Zhang J., Wan W., He Z., Liu J., Liu K., Ren S., Ge L., Du X. (2021). Static-Dynamic Fluorescence Patterns Based on Photodynamic Disulfide Reactions for Versatile Information Storage. Small.

[12] Zhou Y., Zhan P., Ren M., Zheng G., Dai K., Mi L., Liu C., Shen C. (2019). Significant Stretchability Enhancement of a Crack-Based Strain Sensor Combined with High Sensitivity and Superior Durability for Motion Monitoring. Acs Appl. Mater. Interfaces.

[13] Jia F., Huang F., Ouyang S., Cai C., Xu Z., Wu C., Ma Y., Wang M. (2016). Design of photoactive hybrid based intelligent photodetectors for identifying the detected wavelength. J. Mater. Chem. C.

[14] Brighenti R., Cosma M.P. (2020). Swelling mechanism in smart polymers responsive to mechano-chemical stimuli. J. Mech. Phys. Solids.

[15] Zhao X., Wang L.-Y., Tang C.-Y., Zha X.-J., Liu Y., Su B.-H., Ke K., Bao R.-Y., Yang M.-B., Yang W. (2020). Smart Ti3C2Tx MXene Fabric with Fast Humidity Response and Joule Heating for Healthcare and Medical Therapy Applications. Acs Nano.

[16] Zhou J., Jiang B., Gao C., Zhu K., Xu W., Song D. (2022). Stable, reusable, and rapid response smart pH-responsive cotton fabric based on covalently immobilized with naphthalimide-rhodamine probe. Sens. Actuators B-Chem..

[17] Ozcan M., Cakmakci M., Temizer I. (2020). Smart composites with tunable stress-strain curves. Comput. Mech..

[18] Brighenti R., Li Y., Vernerey F.J. (2020). Smart Polymers for Advanced Applications: A Mechanical Perspective Review. Front. Mater..

[19] Sun D., Cao R., Wu H., Li X., Yu H., Guo L. (2023). Harsh Environmental-Tolerant and High-Performance Triboelectric Nanogenerator Based on Nanofiber/Microsphere Hybrid Membranes. Materials.

[20] Feng Y.Q., Lv M.L., Yang M., Ma W.X., Zhang G., Yu Y.Z., Wu Y.Q., Li H.B., Liu D.Z., Yang Y.S. (2022). Application of New Energy Thermochromic Composite Thermosensitive Materials of Smart Windows in Recent Years. Molecules.

[21] Li J., Xu C., Zhang W.Y., Shi P.P., Ye Q., Fu D.W. (2020). Smart and efficient opto-electronic dual response material based on two-dimensional perovskite crystal/thin film. J. Mater. Chem. C.

[22] Li J.N., Lu X.G., Zhang Y., Wen X.X., Yao K.K., Cheng F., Wang D.C., Ke X.Q., Zeng H., Yang S. (2022). Dynamic Refractive Index-Matching for Adaptive Thermoresponsive Smart Windows. Small.

[23] Wang C., Jiang X., Cui P., Sheng M., Gong X., Zhang L., Fu S. (2021). Multicolor and Multistage Response Electrochromic Color-Memory Wearable Smart Textile and Flexible Display. Acs Appl. Mater. Interfaces.

[24] Li J.N., Lu X.G., Zhang Y., Cheng F., Li Y.L., Wen X.X., Yang S. (2020). Transmittance Tunable Smart Window Based on Magnetically Responsive 1D Nanochains. Acs Appl. Mater. Interfaces.

[25] Hou Z.-L., Ma X., Zhang J., Li C., Wang Y., Cao M. (2022). Fascinating Electrical Transport Behavior of Topological Insulator Bi_2_Te_3_ Nanorods: Toward Electrically Responsive Smart Materials. Small.

[26] Liu X., Li Y., Sun X., Tang W., Deng G., Liu Y., Song Z., Yu Y., Yu R., Dai L. (2021). Off/on switchable smart electromagnetic interference shielding aerogel. Matter.

[27] Saqib Q.M., Shaukat R.A., Chougale M.Y., Khan M.U., Kim J., Bae J. (2022). Particle triboelectric nanogenerator (P-TENG). Nano Energy.

[28] Zhang H., Fan T.J., Chen W., Li Y.C., Wang B. (2020). Recent advances of two-dimensional materials in smart drug delivery nano-systems. Bioact. Mater..

[29] Xiao D., Zheng M.-T., Wu F.-J. (2023). A bio-inspired self-assembled asymmetrical supramolecular film for highly-sensitive fire warning, solvent response, and smart switching. Chem. Eng. J..

[30] Mrinalini M., Prasanthkumar S. (2019). Recent Advances on Stimuli-Responsive Smart Materials and their Applications. Chempluschem.

[31] Fan F.-R., Tian Z.-Q., Wang Z.L. (2012). Flexible triboelectric generator!. Nano Energy.

[32] Xing F., Jie Y., Cao X., Li T., Wang N. (2017). Natural triboelectric nanogenerator based on soles for harvesting low-frequency walking energy. Nano Energy.

[33] Jie Y., Jia X.T., Zou J.D., Chen Y.D., Wang N., Wang Z.L., Cao X. (2018). Natural Leaf Made Triboelectric Nanogenerator for Harvesting Environmental Mechanical Energy. Adv. Energy Mater..

[34] Wang N., Zou J.D., Yang Y.X., Li X.Y., Guo Y.L., Jiang C., Jia X.T., Cao X. (2019). Kelp-inspired biomimetic triboelectric nanogenerator boosts wave energy harvesting. Nano Energy.

[35] Guo Y.L., Chen Y.D., Ma J.M., Zhu H.R., Cao X., Wang N., Wang Z.L. (2019). Harvesting wind energy: A hybridized design of pinwheel by coupling triboelectrification and electromagnetic induction effects. Nano Energy.

[36] Liu Y., Sun N., Liu J., Wen Z., Sun X., Lee S.-T., Sun B. (2018). Integrating a Silicon Solar Cell with a Triboelectric Nanogenerator via a Mutual Electrode for Harvesting Energy from Sunlight and Raindrops. Acs Nano.

[37] Yan L.X., Mi Y.J., Lu Y., Qin Q.H., Wang X.Q., Meng J.J., Liu F., Wang N., Cao X. (2022). Weaved piezoresistive triboelectric nanogenerator for human motion monitoring and gesture recognition. Nano Energy.

[38] Khandelwal G., Chandrasekhar A., Raj N.P.M.J., Kim S.-J. (2019). Metal-Organic Framework: A Novel Material for Triboelectric Nanogenerator-Based Self-Powered Sensors and Systems. Adv. Energy Mater..

[39] Lu Y., Mi Y.J., Wu T., Cao X., Wang N. (2022). From Triboelectric Nanogenerator to Polymer-Based Biosensor: A Review. Biosensors.

[40] Dong L., Wang M., Wu J., Zhu C., Shi J., Morikawa H. (2022). Stretchable, Adhesive, Self-Healable, and Conductive Hydrogel-Based Deformable Triboelectric Nanogenerator for Energy Harvesting and Human Motion Sensing. Acs Appl. Mater. Interfaces.

[41] Cheng Y., Wu D., Hao S.F., Jie Y., Cao X., Wang N., Wang Z.L. (2019). Highly stretchable triboelectric tactile sensor for electronic skin. Nano Energy.

[42] Parida K., Thangavel G., Cai G., Zhou X., Park S., Xiong J., Lee P.S. (2019). Extremely stretchable and self-healing conductor based on thermoplastic elastomer for all-three-dimensional printed triboelectric nanogenerator. Nat. Commun..

[43] Zhao K., Wang Z.L., Yang Y. (2016). Self-Powered Wireless Smart Sensor Node Enabled by an Ultrastable, Highly Efficient, and Superhydrophobic-Surface-Based Triboelectric Nanogenerator. Acs Nano.

[44] Md S., Rana S.M.S., Md S., Hye Su S., Selim R.M., Seong Hoon J., Jae Yeong P. (2023). Highly Electronegative V2CTx/Silicone Nanocomposite-Based Serpentine Triboelectric Nanogenerator for Wearable Self-Powered Sensors and Sign Language Interpretation. Adv. Energy Mater..

[45] Chen X., Pu X., Jiang T., Yu A., Xu L., Wang Z.L. (2017). Tunable Optical Modulator by Coupling a Triboelectric Nanogenerator and a Dielectric Elastomer. Adv. Funct. Mater..

[46] Liu S., Li Y., Guo W., Huang X., Xu L., Lai Y.-C., Zhang C., Wu H. (2019). Triboelectric nanogenerators enabled sensing and actuation for robotics. Nano Energy.

[47] Li Y., Chen W., Lu L. (2021). Wearable and Biodegradable Sensors for Human Health Monitoring. ACS Appl. Bio Mater..

[48] Adhikary P., Mahmud M.A.P., Solaiman T., Lin Z. (2022). Recentadvances on biomechanical motion-driven triboelectric nanogenerators for drug delivery. Nano Today.

[49] Dong F., Pang Z., Lin Q., Wang D., Ma X., Song S., Nie S. (2022). Triboelectric nanogenerator enhanced radical generation in a photoelectric catalysis system via pulsed direct-current. Nano Energy.

[50] Wang S., Yuan F., Liu S., Zhou J., Xuan S., Wang Y., Gong X. (2020). A smart triboelectric nanogenerator with tunable rheological and electrical performance for self-powered multi-sensors. J. Mater. Chem. C.

[51] Mi Y.J., Lu Y., Shi Y.L., Zhao Z.Q., Wang X.Q., Meng J.J., Cao X., Wang N. (2023). Biodegradable Polymers in Triboelectric Nanogenerators. Polymers.

[52] Nardekar S.S., Krishnamoorthy K., Manoharan S., Pazhamalai P., Kim S.-J. (2022). Two Faces Under a Hood: Unravelling the Energy Harnessing and Storage Properties of 1T-MoS2 Quantum Sheets for Next-Generation Stand-Alone Energy Systems. ACS Nano.

[53] Meng Q., Zhang M., Tang R., Jin W., Zhang J., Lan Z., Shi S., Shen X., Sun Q. (2022). Stretchable triboelectric nanogenerator with exteroception-visualized multifunctionality. J. Mater. Chem. A.

[54] Dong K., Peng X., Wang Z.L. (2019). Fiber/Fabric-Based Piezoelectric and Triboelectric Nanogenerators for Flexible/Stretchable and Wearable Electronics and Artificial Intelligence. Adv. Mater..

[55] Pang L., Li Z., Zhao Y., Zhang X., Du W., Chen L., Yu A., Zhai J. (2022). Triboelectric Nanogenerator Based on Polyimide/Boron Nitride Nanosheets/Polyimide Nanocomposite Film with Enhanced Electrical Performance. Acs Appl. Electron. Mater..

[56] Ma M., Kang Z., Liao Q., Zhang Q., Gao F., Zhao X., Zhang Z., Zhang Y. (2018). Development, applications, and future directions of triboelectric nanogenerators. Nano Res..

[57] Wen J., He H., Niu C., Rong M., Huang Y., Wu Y. (2022). An improved equivalent capacitance model of the triboelectric nanogenerator incorporating its surface roughness. Nano Energy.

[58] Dharmasena R.D.I.G., Jayawardena K.D.G.I., Mills C.A., Dorey R.A., Silva S.R.P. (2018). A unified theoretical model for Triboelectric Nanogenerators. Nano Energy.

[59] Lee Y., Kang S.G., Jeong J. (2021). Sliding triboelectric nanogenerator with staggered electrodes. Nano Energy.

[60] Wu Y.H., Luo Y., Qu J.K., Daoud W.A., Qi T. (2020). Sustainable and shape-adaptable liquid single-electrode triboelectric nanogenerator for biomechanical energy harvesting. Nano Energy.

[61] Wang Y.Q., Yu X., Yin M.F., Wang J.L., Gao Q., Yu Y., Cheng T.H., Wang Z.L. (2021). Gravity triboelectric nanogenerator for the steady harvesting of natural wind energy. Nano Energy.

[62] You Y., Peng W.L., Xie P., Rong M.Z., Zhang M.Q., Liu D. (2020). Topological rearrangement-derived homogeneous polymer networks capable of reversibly interlocking: From phantom to reality and beyond. Mater. Today.

[63] Edwards C.E.R., Mai D.J., Tang S.C., Olsen B.D. (2020). Molecular anisotropy and rearrangement as mechanisms of toughness and extensibility in entangled physical gels. Phys. Rev. Mater..

[64] Chen C., Chen L., Wu Z., Guo H., Yu W., Du Z., Wang Z.L. (2020). 3D double-faced interlock fabric triboelectric nanogenerator for bio-motion energy harvesting and as self-powered stretching and 3D tactile sensors. Mater. Today.

[65] Zheng L., Dong S., Nie J., Li S., Ren Z., Ma X., Chen X., Li H., Wang Z.L. (2019). Dual-Stimulus Smart Actuator and Robot Hand Based on a Vapor-Responsive PDMS Film and Triboelectric Nanogenerator. Acs Appl. Mater. Interfaces.

[66] Ji S., Shin J., Yoon J., Lim K.-H., Sim G.-D., Lee Y.-S., Kim D.H., Cho H., Park J. (2022). Three-dimensional skin-type triboelectric nanogenerator for detection of two-axis robotic-arm collision. Nano Energy.

[67] Yang D., Kong X., Ni Y., Ren Z., Li S., Nie J., Chen X., Zhang L. (2019). Ionic polymer-metal composites actuator driven by the pulse current signal of triboelectric nanogenerator. Nano Energy.

[68] Du S., Zhou N., Xie G., Chen Y., Suo H., Xu J., Tao J., Zhang L., Zhu J. (2021). Surface-engineered triboelectric nanogenerator patches with drug loading and electrical stimulation capabilities: Toward promoting infected wounds healing. Nano Energy.

[69] Fatma B., Bhunia R., Gupta S., Verma A., Verma V., Garg A. (2019). Maghemite/Polyvinylidene Fluoride Nanocomposite for Transparent, Flexible Triboelectric Nanogenerator and Noncontact Magneto-Triboelectric Nanogenerator. Acs Sustain. Chem. Eng..

[70] Huang L.-b., Xu W., Bai G., Wong M.-C., Yang Z., Hao J. (2016). Wind energy and blue energy harvesting based on magnetic-assisted noncontact triboelectric nanogenerator. Nano Energy.

[71] Zhao X., Nashalian A., Ock I.W., Popoli S., Xu J., Yin J., Tat T., Libanori A., Chen G., Zhou Y. (2022). A Soft Magnetoelastic Generator for Wind-Energy Harvesting. Adv. Mater..

[72] Jana S., Garain S., Ghosh S.K., Sen S., Mandal D. (2016). The preparation ofγ-crystalline non-electrically poled photoluminescant ZnO–PVDF nanocomposite film for wearable nanogenerators. Nanotechnology.

[73] Cheng C.-Y., Chiang Y.-J., Yu H.-F., Hsiao L.-Y., Yeh C.-L., Chang L.-Y., Ho K.-C., Yeh M.-H. (2021). Designing a hybrid type photoelectrochromic device with dual coloring modes for realizing ultrafast response/high optical contrast self-powered smart windows. Nano Energy.

[74] Jao Y.-T., Yang P.-K., Chiu C.-M., Lin Y.-J., Chen S.-W., Choi D., Lin Z.-H. (2018). A textile-based triboelectric nanogenerator with humidity-resistant output characteristic and its applications in self-powered healthcare sensors. Nano Energy.

[75] Ren Z., Ding Y., Nie J., Wang F., Xu L., Lin S., Chen X., Wang Z.L. (2019). Environmental Energy Harvesting Adapting to Different Weather Conditions and Self-Powered Vapor Sensor Based on Humidity-Responsive Triboelectric Nanogenerators. ACS Appl. Mater. Interfaces.

[76] Leyla S., Nassim R., Maryam B., Somayeh F., Azam I., Raheleh M. (2022). Self-Powered Humidity Sensors Based on SnS2 Nanosheets. ACS Appl. Nano Mater..

[77] Li X., Zhang L., Feng Y., Zheng Y., Wu Z., Zhang X., Wang N., Wang D., Zhou F. (2021). Reversible Temperature-Sensitive Liquid–Solid Triboelectrification with Polycaprolactone Material for Wetting Monitoring and Temperature Sensing. Adv. Funct. Mater..

[78] Wang R., Ma J., Ma S., Zhang Q., Li N., Ji M., Jiao T., Cao X. (2022). A biodegradable cellulose-based flame-retardant triboelectric nanogenerator for fire warning. Chem. Eng. J..

[79] Feng M., Kong X., Feng Y., Li X., Luo N., Zhang L., Du C., Wang D. (2022). A New Reversible Thermosensitive Liquid–Solid TENG Based on a P(NIPAM-MMA) Copolymer for Triboelectricity Regulation and Temperature Monitoring. Small.

[80] Liu H., Dong J., Zhou H., Yang X., Xu C., Yao Y., Zhou G., Zhang S., Song Q. (2021). Real-Time Acid Rain Sensor Based on a Triboelectric Nanogenerator Made of a PTFE–PDMS Composite Film. ACS Appl. Electron. Mater..

[81] Li H., Xu F., Guan T., Li Y., Sun J. (2021). Mechanically and environmentally stable triboelectric nanogenerator based on high-strength and anti-compression self-healing ionogel. Nano Energy.

[82] Huang L.-B., Dai X., Sun Z., Wong M.-C., Pang S.-Y., Han J., Zheng Q., Zhao C.-H., Kong J., Hao J. (2021). Environment-resisted flexible high performance triboelectric nanogenerators based on ultrafast self-healing non-drying conductive organohydrogel. Nano Energy.

[83] Wu Z., Chen J., Boukhvalov D.W., Luo Z., Zhu L., Shi Y. (2021). A new triboelectric nanogenerator with excellent electric breakdown self-healing performance. Nano Energy.

[84] Liu R., Kuang X., Deng J., Wang Y.-C., Wang A.C., Ding W., Lai Y.-C., Chen J., Wang P., Lin Z. (2018). Shape Memory Polymers for Body Motion Energy Harvesting and Self-Powered Mechanosensing. Adv. Mater..

[85] Xiong J., Luo H., Gao D., Zhou X., Cui P., Thangavel G., Parida K., Lee P.S. (2019). Self-restoring, waterproof, tunable microstructural shape memory triboelectric nanogenerator for self-powered water temperature sensor. Nano Energy.

[86] Xuan H., Guan Q., Tan H., Zuo H., Sun L., Guo Y., Zhang L., Neisiany R.E., You Z. Light-Controlled Triple-Shape-Memory, High-Permittivity Dynamic Elastomer for Wearable Multifunctional Information Encoding Devices. ACS Nano 2022.16.1.

[87] Van Vleck J.H. (1945). A Survey of the Theory of Ferromagnetism. Rev. Mod. Phys..

[88] Kittel C. (1946). Theory of the Structure of Ferromagnetic Domains in Films and Small Particles. Phys. Rev..

[89] Li Y., Li G., Zhang P., Zhang H., Ren C., Shi X., Cai H., Zhang Y., Wang Y., Guo Z. (2021). Contribution of Ferromagnetic Medium to the Output of Triboelectric Nanogenerators Derived from Maxwell’s Equations. Adv. Energy Mater..

[90] Hariharan P.S., Pan C.J., Karthikeyan S., Xie D.X., Shinohara A., Yang C.L., Wang L., Anthony S.P. (2020). Solvent vapour induced rare single-crystal-to-single-crystal transformation of stimuli-responsive fluorophore: Solid state fluorescence tuning, switching and role of molecular conformation and substituents. Dye. Pigment..

[91] Hu S.Q., Jiang H.P., Zhu J.Q., Wang J.Q., Wang S.H., Tang J.B., Zhou Z.X., Liu S.J., Shen Y.Q. (2021). Tumor-specific fluorescence activation of rhodamine isothiocyanate derivatives. J. Control. Release.

[92] Kazem-Rostami M., Akhmedov N.G., Faramarzi S. (2019). Molecular lambda shape light-driven dual switches: Spectroscopic and computational studies of the photoisomerization of bisazo Troger base analogs. J. Mol. Struct..

[93] Luo Y.Y., Zhang Z.X., Su C.Y., Zhang W.Y., Shi P.P., Ye Q., Fu D.W. (2020). Tunable optoelectronic response multifunctional materials: Exploring switching and photoluminescence integrated in flexible thin films/crystals. J. Mater. Chem. C.

[94] Liu Y.Q., Li E.L., Yan Y.J., Lin Z.N., Chen Q.Z., Wang X.M., Shan L.T., Chen H.P., Guo T.L. (2021). A one-structure-layer PDMS/Mxenes based stretchable triboelectric nanogenerator for simultaneously harvesting mechanical and light energy. Nano Energy.

[95] Bi S.C., Feng C., Wang M.Y., Kong M., Liu Y., Cheng X.J., Wang X.P., Chen X.G. (2020). Temperature responsive self-assembled hydroxybutyl chitosan nanohydrogel based on homogeneous reaction for smart window. Carbohydr. Polym..

[96] Jochum F.D., Theato P. (2012). Temperature- and light-responsive smart polymer materials †. Chem. Soc. Rev..

[97] Dhamecha D., Le D., Chakravarty T., Perera K., Dutta A., Menon J.U. (2021). Fabrication of PNIPAm-based thermoresponsive hydrogel microwell arrays for tumor spheroid formation. Mater. Sci. Eng. C-Mater. Biol. Appl..

[98] Li C., Li M., Ni Z.S., Guan Q.W., Blackman B.R.K., Saiz E. (2021). Stimuli-responsive surfaces for switchable wettability and adhesion. J. R. Soc. Interface.

[99] Sun J., Wang Z., Cao A., Sheng R. (2019). Synthesis of crosslinkable diblock terpolymers PDPA-b-P(NMS-co-OEG) and preparation of shell-crosslinked pH/redox-dual responsive micelles as smart nanomaterials †. RSC Adv..

[100] Kocak G., Tuncer C., Bütün V. (2016). pH-Responsive polymers. Polym. Chem..

[101] Li C., Guo H., Wu Z., Wang P., Zhang D., Sun Y. (2023). Self-Healable Triboelectric Nanogenerators: Marriage between Self-Healing Polymer Chemistry and Triboelectric Devices. Adv. Funct. Mater..

[102] Liu P., Sun N., Mi Y., Luo X., Dong X., Cai J., Jia X., Ramos M.A., Hu T.S., Xu Q. (2021). Ultra-low CNTs filled high-performance fast self-healing triboelectric nanogenerators for wearable electronics. Compos. Sci. Technol..

[103] Korde J.M., Kandasubramanian B. (2020). Naturally biomimicked smart shape memory hydrogels for biomedical functions. Chem. Eng. J..

[104] Delaey J., Dubruel P., Van Vlierberghe S. (2020). Shape-Memory Polymers for Biomedical Applications. Adv. Funct. Mater..

[105] Xu W., Wong M.C., Guo Q.Y., Jia T.Z., Hao J.H. (2019). Healable and shape-memory dual functional polymers for reliable and multipurpose mechanical energy harvesting devices. J. Mater. Chem. A.

[106] Lee J.H., Hinchet R., Kim S.K., Kim S., Kim S.W. (2015). Shape memory polymer-based self-healing triboelectric nanogenerator. Energy Environ. Sci..

[107] Xiang Z., Zekun L., Wenwen D., Yilin Z., Wei W., Linlin P., Li C., Aifang Y., Junyi Z. (2022). Self-powered triboelectric-mechanoluminescent electronic skin for detecting and differentiating multiple mechanical stimuli. Nano Energy.

[108] Yang Y., Lin L., Zhang Y., Jing Q., Hou T.-C., Wang Z.L. (2012). Self-Powered Magnetic Sensor Based on a Triboelectric Nanogenerator. ACS Nano.

[109] Wan D., Ma N., Zhao T., Cui X., Wang Z., Zhang H., Zhuo K. (2021). Magnetorheological Elastomer-Based Self-Powered Triboelectric Nanosensor for Monitoring Magnetic Field. Nanomaterials.

[110] Hajra S., Vivekananthan V., Sahu M., Khandelwal G., Joseph Raj N.P.M., Kim S.-J. (2021). Triboelectric nanogenerator using multiferroic materials: An approach for energy harvesting and self-powered magnetic field detection. Nano Energy.

[111] Wang P., Zhang S., Zhang L., Wang L., Xue H., Wang Z.L. (2020). Non -contact and liquid-liquid interfacing triboelectric nanogenerator for self-powered water/liquid level sensing. Nano Energy.

[112] Ahmed A., Hassan I., Mosa I.M., Elsanadid E., Sharafeldin M., Rusling J.F., Ren S. (2019). An Ultra-Shapeable, Smart Sensing Platform Based on a Multimodal Ferrofluid-Infused Surface. Adv. Mater..

[113] Ma C., Yuan Q., Du H., Ma M.-G., Si C., Wan P. (2020). Multiresponsive MXene (Ti3C2Tx)-Decorated Textiles for Wearable Thermal Management and Human Motion Monitoring. ACS Appl. Mater. Interfaces.

[114] Li W., Lu L., Kottapalli A.G.P., Pei Y. (2022). Bioinspired sweat-resistant wearable triboelectric nanogenerator for movement monitoring during exercise. Nano Energy.

[115] Sheng F.F., Yi J., Shen S., Cheng R.W., Ning C., Ma L.Y., Peng X., Deng W., Dong K., Wang Z.L. (2021). Self-Powered Smart Arm Training Band Sensor Based on Extremely Stretchable Hydrogel Conductors. Acs Appl. Mater. Interfaces.

[116] Song W.X., Gan B.H., Jiang T., Zhang Y., Yu A.F., Yuan H.T., Chen N., Sun C.W., Wang Z.L. (2016). Nanopillar Arrayed Triboelectric Nanogenerator as a Self-Powered Sensitive Sensor for a Sleep Monitoring System. Acs Nano.

[117] Kou H.Y., Wang H.M., Cheng R.W., Liao Y.J., Shi X., Luo J.J., Li D., Wang Z.L. (2022). Smart Pillow Based on Flexible and Breathable Triboelectric Nanogenerator Arrays for Head Movement Monitoring during Sleep. Acs Appl. Mater. Interfaces.

[118] Lai Y.-C., Deng J., Liu R., Hsiao Y.-C., Zhang S.L., Peng W., Wu H.-M., Wang X., Wang Z.L. (2018). Actively Perceiving and Responsive Soft Robots Enabled by Self-Powered, Highly Extensible, and Highly Sensitive Triboelectric Proximity- and Pressure-Sensing Skins. Adv. Mater..

[119] Jin X., Shi Y., Yuan Z., Huo X., Wu Z., Wang Z.L. (2022). Bio-inspired soft actuator with contact feedback based on photothermal effect and triboelectric nanogenerator. Nano Energy.

[120] Ihor S., Sotiria D.P., Antonios T. (2022). Recent Advances in Energy Harvesting from the Human Body for Biomedical Applications. Energies.

[121] Ebtesam Abdullah A.-S., Meneerah Abdulrahman A., Tahani M.A., Hussah Abdullah A., Galyah Mohammed A., Bayan S., Atheel A., Fadwa Mohammed A., Reham Khalid A., Jamilah Naif A. (2022). Nanogenerator-Based Sensors for Energy Harvesting From Cardiac Contraction. Front. Energy Res..

[122] Yuan R., Yang N., Fan S., Huang Y., You D., Wang J., Zhang Q., Chu C., Chen Z., Liu L. (2021). Biomechanical Motion-Activated Endogenous Wound Healing through LBL Self-Powered Nanocomposite Repairer with pH-Responsive Anti-Inflammatory Effect. Small.

[123] Chen S.W., Huang S.M., Wu H.S., Pan W.P., Wei S.M., Peng C.W., Ni I.C., Murti B.T., Tsai M.L., Wu C.I. (2022). A Facile, Fabric Compatible, and Flexible Borophene Nanocomposites for Self-Powered Smart Assistive and Wound Healing Applications. Adv. Sci..

[124] Ping C., Qiong W., Xiao W., Ming Y., Changlu L., Chao X., Bin H., Jiexiong F., Zhiqiang L. (2021). Wireless electrical stimulation of the vagus nerves by ultrasound-responsive programmable hydrogel nanogenerators for anti-inflammatory therapy in sepsis. Nano Energy.

